# With or without you: The paradoxical role of identification in predicting joint and ingroup collective action in intergroup conflict

**DOI:** 10.1002/ejsp.2677

**Published:** 2020-06-23

**Authors:** Siwar Hasan‐Aslih, Eric Shuman, Ruthie Pliskin, Martijn van Zomeren, Tamar Saguy, Eran Halperin

**Affiliations:** ^1^ The Hebrew University of Jerusalem Jerusalem Israel; ^2^ University of Groningen Groningen The Netherlands; ^3^ Leiden University Leiden The Netherlands; ^4^ Interdisciplinary Center Herzliya (IDC) Herzliya Israel

**Keywords:** collective action, identification, intergroup conflict, joint action

## Abstract

While we have a rich understanding of the motivations of disadvantaged group members to act collectively with their group, especially the important role played by identification, we know less about the disadvantaged's motivations to engage in joint action with the advantaged. This research examines the role of identification in predicting joint and ingroup collective action in intergroup conflicts. Since joint action inherently diffuses the perception of “us versus them”, we propose that identification predicts ingroup action, but not joint action. We also examine conflict intensity as a moderator, and examine how changing identification is linked to change in support for joint action. We test these hypotheses in a three‐wave longitudinal study in the Palestinian–Israeli conflict. Results support our hypotheses, demonstrating that identification positively predicts ingroup action but not necessarily joint action, and that when conflict intensifies, changes in identification are negatively related to joint action with outgroup members.

## 1 INTRODUCTION

Palestinians resisting the Israeli occupation and black Americans protesting state violence in the United States are examples of historically disadvantaged groups seeking to overcome prolonged oppression and injustice. Both groups sometimes engage in collective action along with allies from the advantaged group. This joint action may have considerable potential to promote social change, but there is a need to understand when the disadvantaged will be willing and motivated to participate in such action. While research has given us rich descriptions of the motivations of disadvantaged group members to act collectively with other members of their group (ingroup action, see Van Zomeren, Postmes, & Spears, 2008) and the motivations of advantaged groups to act on behalf of the disadvantaged (solidarity based action, see Saab, Tausch, Spears, & Cheung, 2015; Van Zomeren, Postmes, Spears, & Bettache, 2011), it is unclear if these motivations also apply to joint action. Indeed, joint collective action requires members of the disadvantaged group to negotiate between intergroup tension and harmony, that is, between the orientation to characterize the outgroup as the oppressor and the orientation to soften intergroup disparity and accept outgroup members as allies (see Saguy, Tausch, Dovidio, Pratto, & Singh, 2011). Thus, the psychology of joint collective action likely differs from that of other common forms of collective action, particularly when the groups have a history of conflict. For example, identification with the disadvantaged ingroup has been identified as a key variable driving collective action among the disadvantaged (Simon et al., 1998; Sturmer & Simon, 2004), but it is unclear whether this can be generalized to joint action. The aim of the current research is to examine the potentially complex motivational role of ingroup identification among the disadvantaged in predicting joint collective action in the context of intergroup conflict.

Specifically, we compare the relationship between identification and joint action to the relationship between identification and collective action by the ingroup alone (hereafter, “ingroup action”). As conflict intensity may impact the intergroup dynamics at the heart of joint action, we also explore how the relationship changes as a function of fluctuations in conflict intensity over time. We do this in a three‐wave longitudinal study among Palestinians in the context of the Palestinian–Israeli conflict, which went through phases of escalation and de‐escalation throughout the study's duration. We propose that identification would predict ingroup collective action but not necessarily joint collective action. The reason for this is that ingroup collective action resonates more with “us” and with the perception that the ingroup shares the same grievances and fate, whereas joint action diffuses the ingroup and “us” comes to include also (some of) “them”, who are to blame for the ingroup's grievances. Thus, we propose that as a result conflict escalation, situational increases in identification may decrease joint action tendencies. This increase in the salience of group identities could set any collaboration with the outgroup in opposition to the identity and the values of the ingroup. Thus, the more one identifies with the ingroup, the less he/she would want to work together with the advantaged outgroup, even in the context of fighting for change.

### 1.1 Group identification as an antecedent of ingroup collective action

Collective action has been conceptualized in the literature as an event in which individuals, in their capacity as group members, engage in action “directed at improving the conditions of the group as a whole” (Wright, Taylor, & Moghaddam, 1990, p. 995). Taking this definition into account, a large body of literature has examined the psychological processes underpinning collective action (e.g., Kelly & Kelly, 1994; Klandermans, 1997; Sturmer & Simon, 2004; Van Zomeren et al., 2008), demonstrating the importance of four core motivations: emotions associated with perceptions of injustice (e.g., anger), efficacy beliefs about the group's ability to achieve its goals, a sense of identification with the group, and moral convictions (Van Zomeren et al., 2011; Van Zomeren, Spears, Fischer, & Leach, 2004). The vast majority of this research has focused on non‐violent or normative forms of collective action, with more recent work considering predictors of other forms of action and finding, for example, that more radical strategies are predicted by contempt or hatred (Shuman, Cohen‐Chen, Hirsch‐Hoefler, & Halperin, 2016; Tausch et al., 2011). The evidence that non‐violent and violent collective action could be explained by distinct emotions notwithstanding, integrative psychological perspectives on collective action such as the Social Identity Model of Collective Action (SIMCA, Van Zomeren et al., 2008) suggest that identification with the disadvantaged ingroup is a strong predictor of collective action that also informs and amplifies feelings of injustice and efficacy—a contention well in line with the definition presented above, whereby potential benefits to the ingroup are the main target of any collective action.

### 1.2 Why group identification may not predict joint collective action

However, the existing literature offers little insight into the role of social identity when the view of “us versus them” conflicts with the potential benefits of collective action, such as when disadvantaged group members act jointly with advantaged‐outgroup members. We define joint collective action as any action undertaken by members of the disadvantaged and advantaged group together for the purpose of promoting social change. The term “joint” signifies that this action is differentiated from other related forms such as solidarity‐based action (Subašić, Reynolds, & Turner, 2008), which can be undertaken by advantaged group members without the presence of the disadvantaged. This collaboration between disadvantaged and advantaged group members is potentially meaningful as it can bolster the struggle of the disadvantaged by increasing their access to resources and decision‐making processes and communicating to the public norms against inequality and injustice (Louis, 2009).

Consistent with the notion that strong identification with the ingroup should translate in theory, into any collective action that aims to advance the goals of the ingroup, hypothetically it should also be positively linked to joint collective action. Despite such potential, joint collective action appears to be less common than collective action that involves the disadvantaged group alone, and in certain cases it can be controversial, especially when decades of oppression and injustice underlie the conflict between groups. The uniqueness of joint action lies in its conflicting components, namely the collective action component and the component of intergroup contact between groups that vary in power and status, and may even be enemies. On the one hand, collective action aims to change the status quo and emerges from perceptions of intergroup differences and strong identification with the ingroup. On the other hand, however, cooperative intergroup encounters are often structured to de‐emphasize such differences and highlight commonalities between the groups (Becker, Wright, Lubensky, & Zhou, 2013; Saguy, 2018; Wright & Lubensky, 2009). Thus, for disadvantaged group members, joint action encompasses inherent conflicts between their perception of the outgroup as oppressive and the perception of outgroup members as allies, and between their ingroup identity and the common identity that can emanate from collaborating with the outgroup.

This dynamic raises the question of whether joint collective action—like ingroup collective action—is predicted by strong ingroup identification. Specifically, it is plausible that for disadvantaged group members, particularly in the context of intergroup conflict, the need to differentiate their group from the oppressor is so meaningful that it makes it hard to think of outgroup members as partners. In addition, in the context of intergroup conflicts there may be very little trust between the two groups, making such partnership challenging (Çelebi, Verkuyten, Köse, & Maliepaard, 2014). If so, by creating a shared front, joint action involves some decategorization of the distinction between us—the disadvantaged—and them—the advantaged— and may thus be unattractive to strong identifiers. This is consistent with previous research suggesting that strong identification is associated with motivations for intergroup differentiation and therefore can undermine willingness to cooperate with the outgroup (Brewer, 1996; Kelly, 1988; Saguy, Dovidio, & Pratto, 2008). Furthermore, because joint action involves cooperative contact with the outgroup, it may be unattractive to strong identifiers (Ron, Solomon, Halperin, & Saguy, 2017). Accordingly, the present research seeks to illuminate the complex role of ingroup identification in predicting joint collective action tendencies among the disadvantaged.

The question of how a strong identity relates to joint action is particularly important when we consider the dynamics of conflict across time. Conflicts between groups do not develop along a unidirectional path, but rather pass through phases of escalation and de‐escalation (Kriesberg, 2007). These changes are also meaningful when considering levels of ingroup identification. The salience of group identities likely increases during conflict escalation because group members feel that their identity is threatened, which contributes to the polarization of identities and feeds a climate of distrust, defensiveness, and hostility (Fisher, 2006; Klandermans, 2014). In keeping with this, during phases of escalation groups are mobilized for intergroup struggle and are more willing to undertake confrontational actions in an attempt to protect the ingroup (Kriesberg, 2007). Thus, intergroup boundaries become firmer, leaving little freedom to blur intergroup distinctions. Under such circumstances, disadvantaged group members may come to see cooperation as opposite to what they stand for, so that strong identification would not promote joint action, and situational increases in identification may even decrease willingness for joint action.

### 1.3 The current research

Previous work has emphasized ingroup identification as a driving force for ingroup collective action. Joint action, on the other hand, blurs intergroup boundaries, and involves cooperation with outgroup members in a conflict, and may therefore be met with resistance in contexts in which such boundaries are meaningful. The current research aims to investigate the link between ingroup identification and joint action tendencies, particularly in the context of intergroup conflict, which heightens the distinction between ingroup and outgroup. We also consider conflict escalation as a potential moderator of the relationship between identification and joint collective action by examining how these relationships change over time. This allows us to explore two different types of effects: both how general levels of identification predict joint action over time, and how situational changes in identification within subjects relate to change in willingness for joint action. We propose that in the context of intergroup conflict, general group identification does not predict willingness to undertake joint action with outgroup members, and we expect that this relationship may even become negative during times of conflict escalation. In addition, situational fluctuations in identification should be negatively related to joint action, such that if a person's identification is heightened their willingness for joint action decreases.

To understand the complex relationship between identification and joint action, we analyzed data from a three‐wave study we conducted in the context of the Palestinian–Israeli conflict, in which conflict resolution has been elusive for decades. The origin of this conflict can be traced back to the growth of the Zionist movement and the 1948 war (Kelman, 1999). These led to the establishment of a Jewish state in Palestine and the displacement of 80% of the Palestinian population (referred to as the *Nakba*, meaning catastrophe), which was countered by a rise in the Palestinian national identity and resistance. During the 1967 war, Israel seized what is now known as the Occupied Palestinian territories, a turning point that shaped the conflict and Palestinian struggle for liberation ever since (Badil, 2004; Khalidi, 1997). The lasting experience of Palestinians with oppression and injustice has motivated both strong identification and different forms of collective action, including popular and armed resistance, but also joint Palestinian–Israeli actions. Despite its potential value to advancing social change, such joint actions have become less and less common with the Palestinian–Israeli peace process reaching a deadlock. Palestine/Israel thus offers a unique context in which to examine joint collective action among a disadvantaged group experiencing prolonged oppression and a conflict that continuously undergoes phases of escalation and de‐escalation.

## 2 METHOD

### 2.1 Participants and procedure

Participants were Palestinians living in the city of Ramallah and the surrounding areas in the West Bank, who were recruited by a local survey company (Near East Consulting) for face‐to‐face interviews. As online polling companies are not available in this region, and due to the sensitive socio‐political topics of the study, recruiting participants and maintaining their participation over a long period of time posed a significant challenge. Our past research experiences in the West Bank encountered distrust and lack of cooperation by many of the Palestinians approached, who expressed concerns and fear of being subject to political persecution by the Israeli army or the Palestinian Authority—concerns that are common to marginalized and oppressed groups. To overcome these challenges, the survey company employed convenience sampling, in which survey personnel recruited people in their social network, alongside efforts to obtain population representation consistent with statistics from the Palestinian Central Bureau of Statistics. The final sample was generally similar in demographics to the overall population, with similar gender and age distributions (see Table S1 in Supporting Information).

We administered the first wave in May 2018, during a period of relative calm, allowing us to assess all variables at baseline levels. Four hundred and fifty participants (51% women; ages 18–70, *M*_age_ = 33.9) completed the first wave (T1). The sample size was determined by a generic power analysis, as this survey would be used for a number of research projects. We aimed to be able to detect small changes across time points (*d* = 0.2) with high sensitivity (95% power at the *p* = .01 level). A power analysis conducted in G*Power indicated that a sample of 449 was required. We collected data for a second wave (T2) 7 months later, during a period of escalation following two drive‐by shootings carried out by Palestinians that targeted Israeli soldiers and settlers near illegal Israeli settlements. The Israeli army imposed a military closure on Ramallah, raiding residential neighborhoods and shutting down major checkpoints between it and surrounding cities—all of which meant the escalation significantly affected our participants. Finally, we administered a third wave 9 months later, during another period of “calm”.

Our choice of data collection methodology proved effective, as participant attrition was minimal. The vast majority of participants completed all three waves (*n = *420, 50% women, *M*_age_ = 33.7), and only these were included in the final analyses. In all waves, after obtaining their informed consent,^1^ the interviewer read to participants the questions and recorded their answers. Each interview lasted around 40–60 min, and each participant received an anonymized identification code, allowing us to match responses across the waves.

### 2.2 Measures

Our primary variables of interest (identification and joint and ingroup collective action intentions) as well as other relevant constructs from the SIMCA model (anger and efficacy) were measured at all time points. In addition, we included a measure of tolerance for violence against the other side across time as an indicator of conflict escalation. During periods of escalation we would expect participants to become more supportive and tolerant of violence, even if they themselves are not actively engaged in violent action. Demographic variables were measured only at T1. Given the small number of missing data points (no variable contained more than 10% missing values), we used a pairwise deletion technique to handle missing data in our analyses, which is preferred over more complex imputation procedures in this case (Newman, 2014).

This study was a part of a large‐scale survey that examined a number of research questions and thus it included additional measures that were not analyzed in the current study. The data obtained from these additional measures will be used in future publications.^2^ We report the full list of measures in Appendix S1. Unless otherwise indicated, all items reported below were measured on a 6‐point Likert‐type scale anchored 1 (*Not at all*) and 6 (*To a very large extent*).

#### 2.2.1 Group identification

Participants were asked to indicate the extent to which they agreed with the following statements: “Being Palestinian is an important part of my identity” and “I identify with other Palestinians” (T1 *r* = .82, T2 *r* = .84, T3 *r* = .66) (adapted from Roccas, Sagiv, Schwartz, Halevy, & Eidelson, 2008; Van Zomeren et al., 2004).

#### 2.2.2 Collective action intentions

Participants were asked about their willingness to engage in ingroup collective action: “Participating in demonstrations against the occupation”, “Participating in sit‐ins against the occupation”, “Acting within social political movements against the occupation”, “Supporting the political and economic boycott of Israel”, and “Organizing campaigns targeting the public and merchants to boycott Israeli products” (T1 α = 0.97, T2 α = 0.99, T3 α = 0.96) (adapted from Van Zomeren et al., 2004, and Tausch et al., 2011); and in joint collective action: “Participating in joint Palestinian–Israeli peace initiatives” and “Participating in joint Palestinian–Israeli demonstrations against the occupation” (T2 *r* = .82, T2 *r* = .84, T3 *r* = .66).

#### 2.2.3 Tolerance of violent action

Participants rated the extent to which they thought violent collective action was acceptable: “To what extent do you think it is understandable that people resort to non‐peaceful means of resistance?”, “To what extent do you think it is legitimate that people resort to non‐peaceful means of resistance?”, and “To what extent to you understand people who engage in armed resistance?” (T1 α = 0.92, T2 α = 0.91, T3 α = 0.90) (Tausch et al., 2011).

#### 2.2.4 Demographics

Participants completed a brief demographic questionnaire in T1. Items included gender, age, education, income, religion, religiosity, and profession.

## 3 RESULTS

**FIGURE 1:**
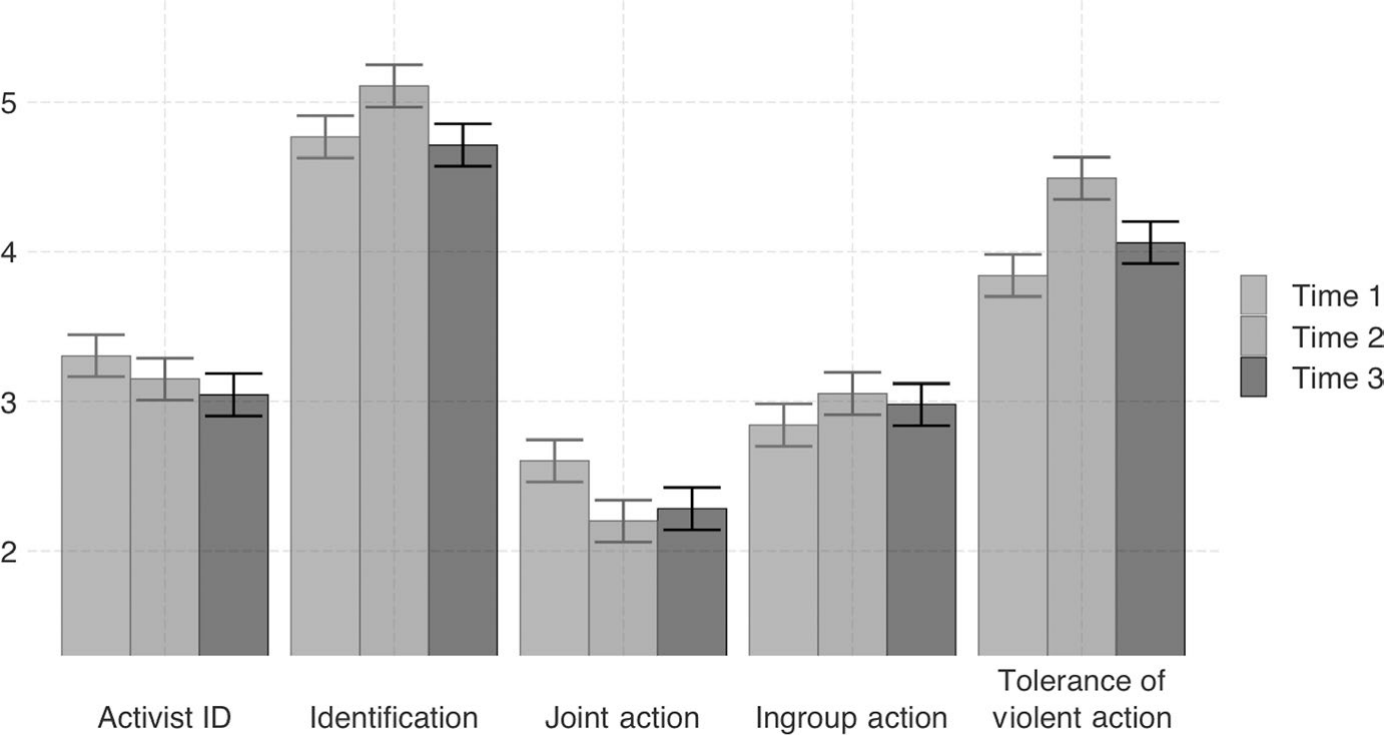
Change in measured variables across time. *Note*. Error bars reflect 95% confidence intervals

The code used to analyze the data can be found at https://osf.io/5xvwe/. We first examined the means of all study variables at each time point (see Table 1; Figure 1) and tested how these varied across time (through the periods of escalation and de‐escalation) using repeated measures analyses of variance (ANOVAs). Bivariate correlations between main study variables at all time points are presented in Appendix S1.

**TABLE 1:** Descriptive statistics for measured variable and changes in these across time

		Time 1 *M* (*SD*)	Time 2 *M* (*SD*)	Time 3 *M* (*SD*)
Group identification	*F*(2, 838) = 18.12, *p* < .001	4.76 (1.28)^a^	5.10 (1.14)^b^	4.71 (1.12)^a^
Ingroup collective action	*F*(2, 838) = 3.58, *p* = .03	2.83 (1.54)^a^	3.05 (1.81)^b^	2.98 (1.73)^b^
Joint collective action	*F*(2, 838) = 13.01, *p* < .001	2.59 (1.44)^a^	2.20 (1.33)^b^	2.29 (1.48)^b^
Tolerance of violent Collective action	*F*(2, 838) = 35.64, *p* < .001	3.85 (1.38)^a^	4.49 (1.31)^b^	4.06 (1.36)^c^

Note

Means in the same row with different superscripts denote significant differences at the *p* < .05 level.

In the first ANOVA, we checked whether our data reflected the escalation generated by the Palestinian attacks and subsequent Israeli military actions that occurred at T2. To reflect an escalation at T2 relative to T1 and T3, we would expect to see a peak in tolerance for violent actions towards Israelis at that time point. Indeed, the analysis shows an increase in tolerance for violence from T1 to T2 followed by a decrease from T2 to T3, which is in line with our understanding of T2 coinciding with a period of heightened escalation. Consistent with our expectations, identification with the ingroup was also significantly higher at T2.

Ingroup collective action also showed an increase from T1 to T2, but remained high at T3, months following the escalation. Conversely, willingness to participate in joint action *decreased* from T1 to T2 and remained low at T3. We also examined the distributions of all variables (see Appendix S1 for violin plots), finding that the skewness for all variables approached an absolute value of 0.5 or higher. We therefore log‐transformed all variables to meet the assumption required for the central regression analyses.^3^

### 3.1 The relationship between identification and collective action across time

We investigated the role of identification in explaining both ingroup and joint action at each time point using a mixed‐model analysis with the lme4 and lmerTest packages in R. Since our primary predictor of interest, that is identification, was measured and also varied across time we employed the methods described by Wang and Maxwell (2015) in order to disentangle within versus between participant effects, by centering and separating identification into within and between subject predictors. If such steps are not taken, the meaning of the effect of identification in the model is unclear as it reflects a mixture of two effects: (a) the effect of a person's general level of identification, which remains constant across situations (between subjects effect) and (b) situationally dependent fluctuations of identification (within subjects effect). To capture the effect of changes in identification, participants’ scores on identification were participant mean centered, and this was entered as a continuous within‐subject predictor. To capture the effect of general levels of identification, participants’ mean identification (across time) was centered to the grand mean and entered as a continuous between‐subjects predictor. In addition, type of action (ingroup vs. joint) and time (T1 vs. T2 vs. T3) were treated as categorical within‐subject variables. Since time was a categorical variable, we dummy‐coded it so that the period of escalation (T2) was the reference variable, as we were particularly interested in how processes at this time point compared to the other two time points. This yielded two dummy variables: one reflecting the difference between the T1 and T2 (escalation), and the second the difference between T3 and T2. All variables other than the dummy variables were mean centered for the interpretability of main effects and two‐way interactions.

On our hypotheses we were primarily interested in two potential interactions. First, we were interested in the interaction between change in identification and type of action, as this would tell us whether situational increases or decreases in levels of identification were linked with increases or decreases in ingroup versus joint action across time. Second, we were interested in the interaction between participants’ general level of identification, type of action, and time. This interaction would give us information about whether general levels of identification predicted ingroup versus joint action differently at times of escalation versus de‐escalation. Below, we focus our discussion of the results on these interactions, with statistics for all other regression coefficients presented in Table 2.

**TABLE 2:** Regression coefficients for the full model predicting collective action

Predictors	Willingness to engage in action				
	Estimates	*SE*	95% CI	Statistic	*p*
Intercept	1.23***	0.02	1.20, 1.26	78.69	<.001
Time D1 (Time 2 vs. Time 1)	0.03	0.02	−0.01, 0.06	1.59	.11
Time D2 (Time 2 vs. Time 3)	−0.02	0.02	−0.05, 0.01	−1.26	.21
Type of action	0.28***	0.02	0.24, 0.33	12.42	<.001
General identification	0.38***	0.09	0.20, 0.56	4.16	<.001
Changes in identification	−0.01	0.04	−0.08, 0.06	−0.30	.76
Time D1 × Type of action	−0.17***	0.03	−0.23, −0.10	−5.14	<.001
Time D2 × Type of action	−0.05	0.03	−0.11, 0.02	−1.45	.15
Time D1 × General identification	0.14	0.09	−0.04, 0.32	1.52	.13
Time D1 × General identification	0.33***	0.09	0.15, 0.51	3.53	<.001
Type of action × General identification	0.66***	0.13	0.40, 0.92	5.04	<.001
Type of action × Changes in identification	0.25***	0.07	0.10, 0.39	3.32	.001
Time D1 × Type of action × General identification	−0.36	0.19	−0.73, 0.00	−1.95	.050
Time D2 × Type of action × General identification	−0.15	0.19	−0.52, 0.21	−0.83	.41

Random effects	
σ^2^	0.11
*τ*_00 id_	0.05
ICC	0.32
*N*_id_	421
Observations	2,508
Marginal *R* ^2^/Conditional *R* ^2^	.136/.408

***p < .001.

**FIGURE 2:**
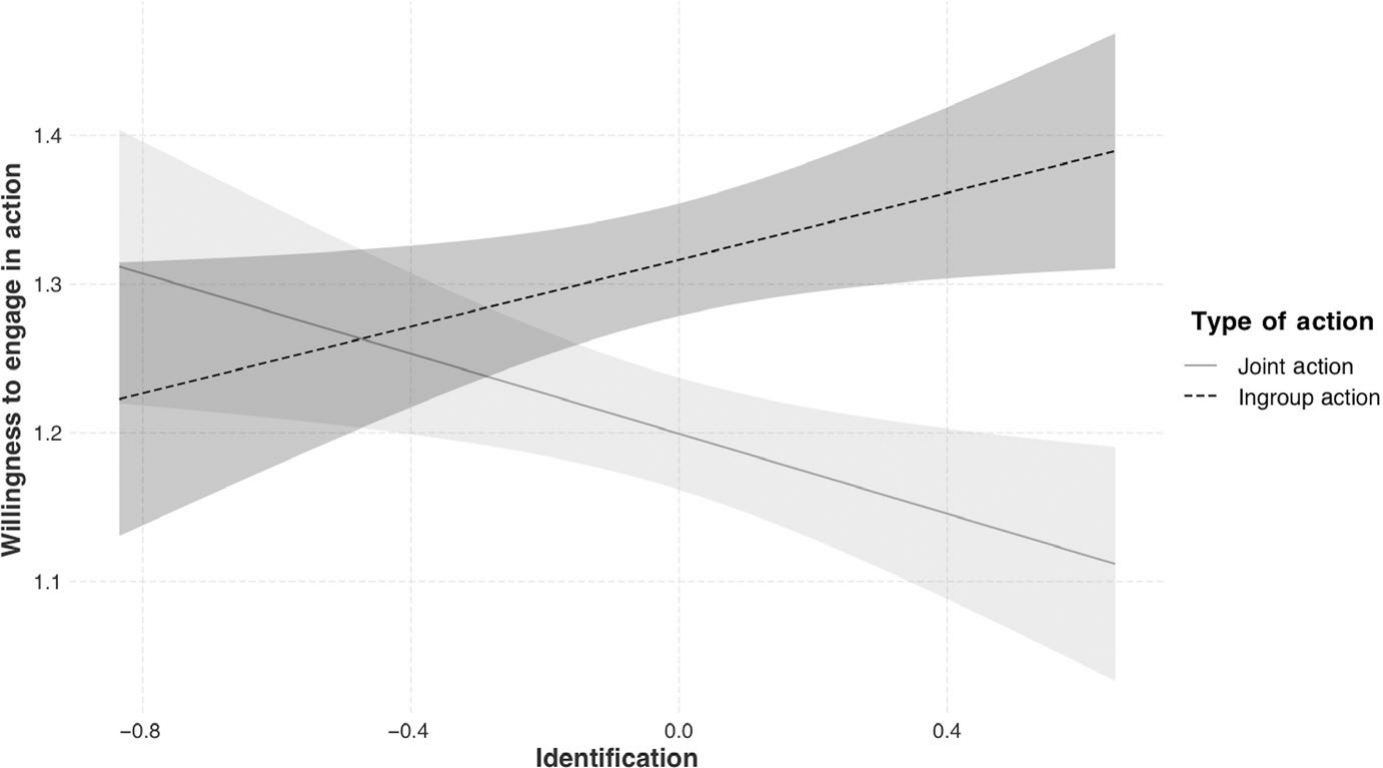
The interaction of change in identification and type of action willingness to partake in collective action across time. *Note*. Shaded areas reflect 95% confidence intervals, and all variables are log transformed

As predicted, the interaction between change in identification and action type was significant, *b* = 0.25, 95% CI [0.10, 0.39], *SE* = 0.07, *t* = 3.32, *p* = .001 (see Figure 2). Simple slopes analyses reveal that changes in identification were positively related to ingroup action across time, meaning that increases in identification were associated with increases in ingroup action, *b* = 0.11, 95% CI [0.01, 0.22], *SE* = 0.05, *t* = 2.13, *p* = .03. However, changes in identification were negatively related to joint action across time, indicating that increases in identification were associated with decreases in joint action, *b* = −0.13, 95% CI [−0.24, −0.03], *SE* = 0.05, *t* = −2.56, *p* = .01.

**FIGURE 3:**
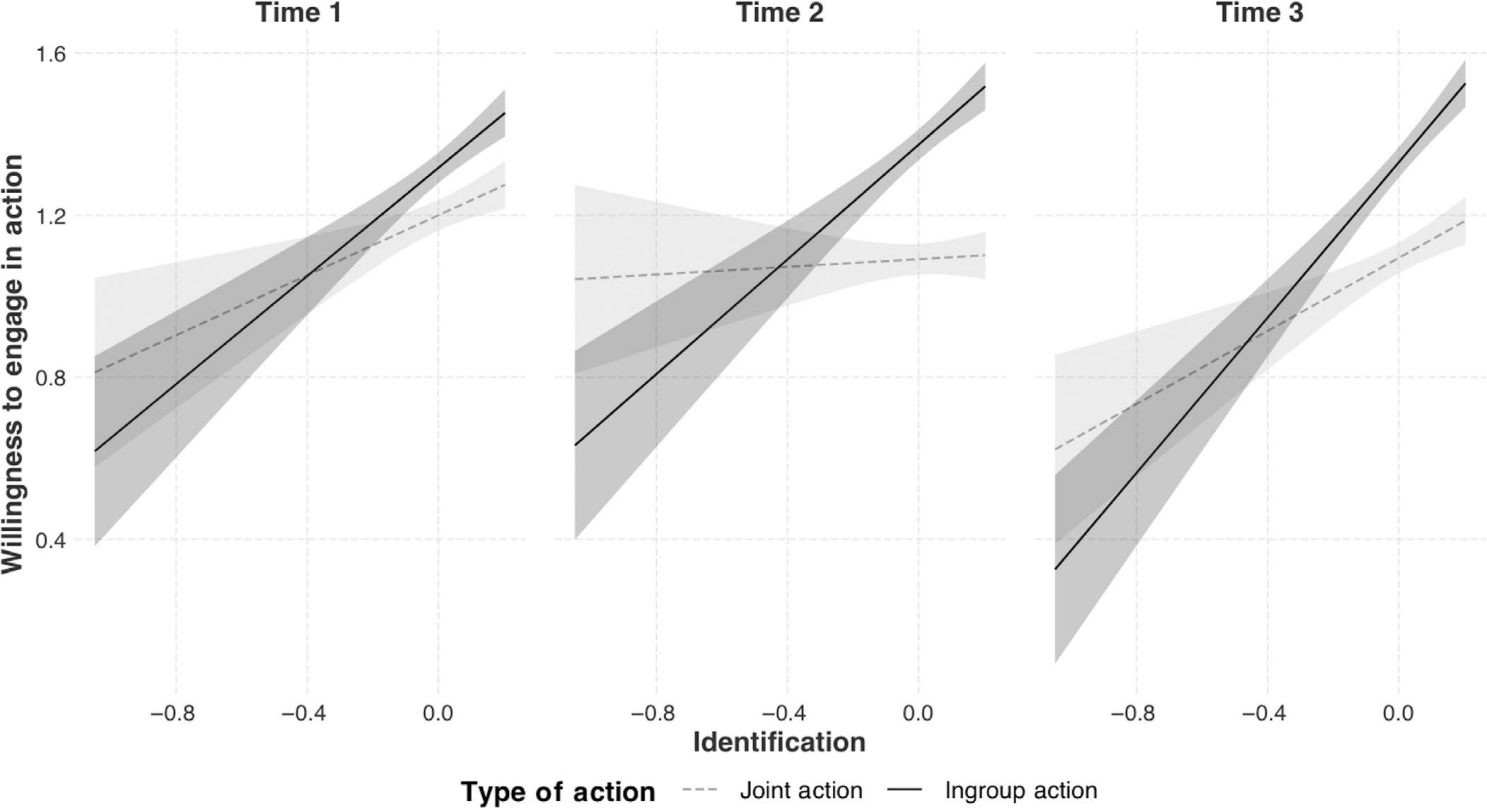
The three‐way interaction of general identification, type of action, and time on willingness to partake in collective action. *Note*. Shaded areas reflect 95% confidence intervals, and all variables are log transformed

In addition, the three‐way interaction between general levels of identification, type of action, and the dummy variable reflecting the comparison between T1 and T2 was marginally significant, *b* = −0.36, 95% CI [−0.73, 0.00], *SE* = 0.19, *t* = −1.95, *p* = .050 (see Figure 3). However, the three‐way interaction between type of action, identification, and the dummy variable reflecting the comparison between T3 and T2 was not significant, *b* = −0.15, 95% CI [−0.54, 0.00], *SE* = 0.19, *t* = −0.83, *p* = .41.^4^ Analysis of simple slopes revealed that at T1 general identification was associated with joint action, *b* = 0.37, 95% CI [0.15, 0.59], *SE* = 0.11, *t* = 3.30, *p* < .001, and it positively predicted ingroup action, *b* = 0.67, 95% CI [0.45, 0.89], *SE* = 0.11, *t* = 5.93, *p* < .001. At T2, general identification still positively predicted ingroup action, *b* = 0.71, 95% CI [0.49, 0.93], *SE* = 0.11, *t* = 6.32, *p* < .001, but was *not* associated with joint action, *b* = 0.05, 95% CI [−0.17, 0.27], *SE* = 0.11, *t* = 0.42, *p* = .67. At T3 the pattern was similar to T1, such that identification positively predicted ingroup action, *b* = 0.96, 95% CI [0.74, 1.18], *SE* = 0.11, *t* = 8.55, *p* < .001, and positively predicted joint action, *b* = 0.45, 95% CI [0.23, 0.67], *SE* = 0.11, *t* = 4.03, *p* < .001.^5^

Because we were particularly interested on the changing effect of identification on joint action over time, we wanted to ensure that the differences between the effects were statistically significant (especially given that the three‐way interaction was marginally significant). Therefore, we ran an additional analysis only using joint action as the dependent variable, in which case the significance of the interaction terms between time and general identification only reflect significant differences in the effect of identification on joint action across time. We ran the same analysis but without ingroup action, and thus without action type as a predictor (see Table 3). The interaction between general levels of identification and the dummy variable reflecting the comparison between T1 and T2 was significant, *b* = 0.32, 95% CI [0.05, 0.59], *SE* = 0.14, *t* = 2.33, *p* = .02, and interaction between general identification and the dummy variable reflecting the comparison between T3 and T2 was also significant, *b* = 0.40, 95% CI [0.13, 0.67], *SE* = 0.14, *t* = 2.94, *p* = .003. Analysis of simple slopes revealed that at T1 general identification was associated with joint action, *b* = 0.47, 95% CI [0.24, 0.69], *SE* = 0.11, *t* = 4.12, *p* < .001, but at T2 was *not* associated with joint action, *b* = 0.06, 95% CI [−0.17, 0.28], *SE* = 0.11, *t* = 0.50, *p* = .62. The T3 results were similar to T1 and general identification positively predicted joint action, *b* = 0.35, 95% CI [0.13, 0.57], *SE* = 0.11, *t* = 3.07, *p* < .001. These significant interactions indicated that there were significant differences in the effect of general identification across time, and that the weaker interactions in the prior model were likely due to the stability of identification's effect on ingroup action across time.

**TABLE 3:** Regression coefficients for the full model predicting joint action only

Predictors	Joint action				
	Estimates	*SE*	95% CI	Statistic	*p*
Intercept	1.09***	0.02	1.05, 1.13	−1.88	<.001
Time D1 (Time 2 vs. Time 1)	0.11***	0.02	0.06, 0.15	4.52	<.001
Time D2 (Time 2 vs. Time 3)	0.00	0.02	−0.04, 0.05	0.12	.90
General Identification	0.05	0.11	−0.17, 0.27	0.42	.67
Changes in Identification	−0.13*	0.06	−0.24, −0.03	−2.45	.01
Time D1 × General Identification	0.32*	0.14	0.05, 0.59	2.33	.02
Time D1 × General Identification	0.40**	0.14	0.13, 0.67	2.94	.003

Random Effects	
σ^2^	0.12
*τ*_00 id_	0.04
ICC	0.26
*N*_id_	421
Observations	1,258
Marginal *R* ^2^/Conditional *R* ^2^	.040/.291

* *p* < .05; **p < .01; ***p < .001.

## 4 DISCUSSION

Identity is an important driving force that motivates people to take action to promote the interests of their group. While identification can facilitate participation in collective action that emphasizes connectedness and shared fate between members of the ingroup, the current research shows the complicated role of identification in predicting action that includes members of the advantaged outgroup in the collective, especially when the disadvantaged are in direct conflict with the advantaged. In this article, we conducted a three‐wave longitudinal study to investigate the relationship between group identification among disadvantaged group members and their joint (vs. ingroup) collective action tendencies in the context of intergroup conflict.

Our results revealed that high identification predicts tendencies for ingroup collective action and for joint collective action, but only during periods of relative calm. During escalation general identification with the ingroup *no longer* predicts joint action. In other words, while strongly identified individuals are more likely to engage in joint action than weakly identified individuals during periods of calm, during escalation individuals are uniformly unwilling to engage in joint action regardless of identification. While we did not find support for our hypothesis that the relationship would become negative during escalation, the relationship did significantly decrease, shifting from a positive to a null relationship. In addition, our results did indicate that situational increases in identification, driven by such escalation, were negatively related to change in joint action. In other words, when conflict events lead individuals to identify more strongly with their group, their motivation for joint action decreases, while their motivation for ingroup action increases. Overall, these results suggest that high identifiers, despite their strong commitment to their group, are not always driven to jointly engage in collective action with outgroup allies. In fact, when conflict exacerbates, their strong identification pulls them away from any cooperation with the outgroup, even if such cooperation is designed to promote social change.

The present research offers an important contribution to the literature on collective action, as it demonstrates that the psychological processes underlying joint collective action may be distinct from those underlying ingroup collective action. Established social identity approaches to collective action have thus far focused on how a strong sense of identification with the group drives motivation to engage in collective action on the behalf of the group (Tajfel & Turner, 1979; Van Zomeren et al., 2008). However, joint action is not just collective action, but also a form of cooperative intergroup contact with outgroup allies. Thus, issues such as lack of trust, ideological differences, and clashes between identities could be barriers to joint action for high identifiers. Our results reflect this complexity: While identification predicted joint action during periods of relative calm, there was no relationship between identification and joint action during escalation, and situational increases in identification decreased joint action.

Acting together with outgroup members is likely to shift focus away from the identity of the disadvantaged and generate a superordinate identity that includes both the disadvantaged and the advantaged. Especially during times of heightened conflict, high identifiers seem to resist cooperating with the outgroup under a common identity. This could stem from concerns that outgroup allies may not be dependable and might retreat from action during more threatening circumstances. This reluctance to cooperate with the advantaged outgroup may indicate that disadvantaged group members believe that inclusion of allies from the outgroup may eventually result in the marginalization of their identity and the domination of the advantaged over the movement. Consistent with this, recent work by Hasan‐Aslih et al. (under review) shows that concerns about normalization of power relations (i.e., obscuring power relations in ways that make them appear normal) may demotivate the disadvantaged from engaging in joint action with outgroup members, particularly when their group identification is high (see also Droogendyk, Wright, Lubensky, & Louis, 2016; Louis, 2009). High identifiers may be concerned that during such times of escalation, joint action may not be aimed at promoting the goals of their disadvantaged group but rather communicating messages of commonality and harmony that can distract from inequality (Saguy, Tausch, Dovidio, Pratto, 2009).

We acknowledge that the current research was conducted in a context of protracted conflict that is characterized by high intensity and violence, thereby engendering extreme disparities between identities. Although theory would suggest that the psychological dynamics of joint collective action should apply to other social contexts as well, future research may investigate the relationship between identification and joint action in additional intergroup conflicts that differ in severity. In addition, despite the evidence that joint action is more weakly predicted by ingroup identification than ingroup collective action, and that this identification may even decrease support for joint action, it is plausible that it is driven by a different social identity that was not considered in our study. Future research could thus examine whether motivation for joint action can be promoted by a superordinate identity that encompasses the identities of both groups.

## CONFLICT OF INTEREST

The authors declare that there are no potential conflicts of interest with respect to the research, authorship, and/or publication of this article.

## ETHICAL APPROVAL

The study was approved by a specific IRB committee set up to oversee the research funded by a grant to the last author.

## TRANSPARENCY STATEMENT

The original data files and code used for data management and analysis can be found at https://osf.io/5xvwe/.

## Supporting information

Supplementary Material

## Footnotes

^1^ The study was approved by a specific IRB committee set up to oversee the research funded by a grant to the last author.

^2^ One paper based on data from only the first two waves of this study has already been published (see Hasan‐Aslih & Shuman et al., 2020).

^3^ We obtain the same results if we use raw values.

^4^ If we add anger and efficacy (the other classical predictors of collective action), as well as demographics, as covariates then these interactions remain at the same or increase in significance level, that is 0.04, and 0.35 respectively.

^5^ It is worth noting that using a different dummy‐coding scheme, which allows for a comparison of T1 and T3, reveals that there is no significant difference in the pattern of results between T1 and T2.
