# Supplementary material for: With or without you: The paradoxical role of identification in predicting joint and ingroup collective action in intergroup conflict
Source: Eur J Soc Psychol. 2020 Jun 23;50(6):1334–43. doi: 10.1002/ejsp.2677 (PMC7754399; doi:10.1002/ejsp.2677)
Supplement: Supplementary file 1 — Supplementary Material [file EJSP-50-1334-s001.docx]

**Supplementary Materials**

**APPENDIX**

Table 1

Sample and Population Demographics

|  | Sample | Population |
| --- | --- | --- |
|  |  |  |
| Gender |  |  |
| Male | 50% | 50% |
| Female | 50% | 50% |
|  |  |  |
| Age |  |  |
| 18-29 | 46.7% | 40% |
| 30-60 | 50.2% | 51% |
| 60+ | 3.1% | 9% |

*Note*. Population statistics are based on data of the Palestinian Central Bureau of Statistics

Table 2

*Means, standard deviations, and correlations with confidence intervals*

| Variable | 1 | 2 | 3 | 4 | 5 | 6 | 7 | 8 |
| --- | --- | --- | --- | --- | --- | --- | --- | --- |
|  |  |  |  |  |  |  |  |  |
| 1. Identification T1 |  |  |  |  |  |  |  |  |
|  |  |  |  |  |  |  |  |  |
| 2. Ingroup CA T1 | .27** |  |  |  |  |  |  |  |
|  |  |  |  |  |  |  |  |  |
| 3. Joint CA T1 | .14** | .70** |  |  |  |  |  |  |
|  |  |  |  |  |  |  |  |  |
| 4. Identification T2 | .36** | .17** | .12* |  |  |  |  |  |
|  |  |  |  |  |  |  |  |  |
| 5. Ingroup CA T2 | .19** | .42** | .28** | .32** |  |  |  |  |
|  |  |  |  |  |  |  |  |  |
| 6. Joint CA T2 | .04 | .19** | .23** | .03 | .27** |  |  |  |
|  |  |  |  |  |  |  |  |  |
| 7. Identification T3 | .22** | .15** | .15** | .16** | .11* | .05 |  |  |
|  |  |  |  |  |  |  |  |  |
| 8. Ingroup CA T3 | .30** | .47** | .38** | .31** | .55** | .11* | .18** |  |
|  |  |  |  |  |  |  |  |  |
| 9. Joint CA T3 | .27** | .37** | .36** | .14** | .19** | .35** | .03 | .39** |
|  |  |  |  |  |  |  |  |  |

*Note.** indicates *p* < .05. ** indicates *p* < .01.

**Figures 1 – 6.** Violin Plots for Main Study Variables


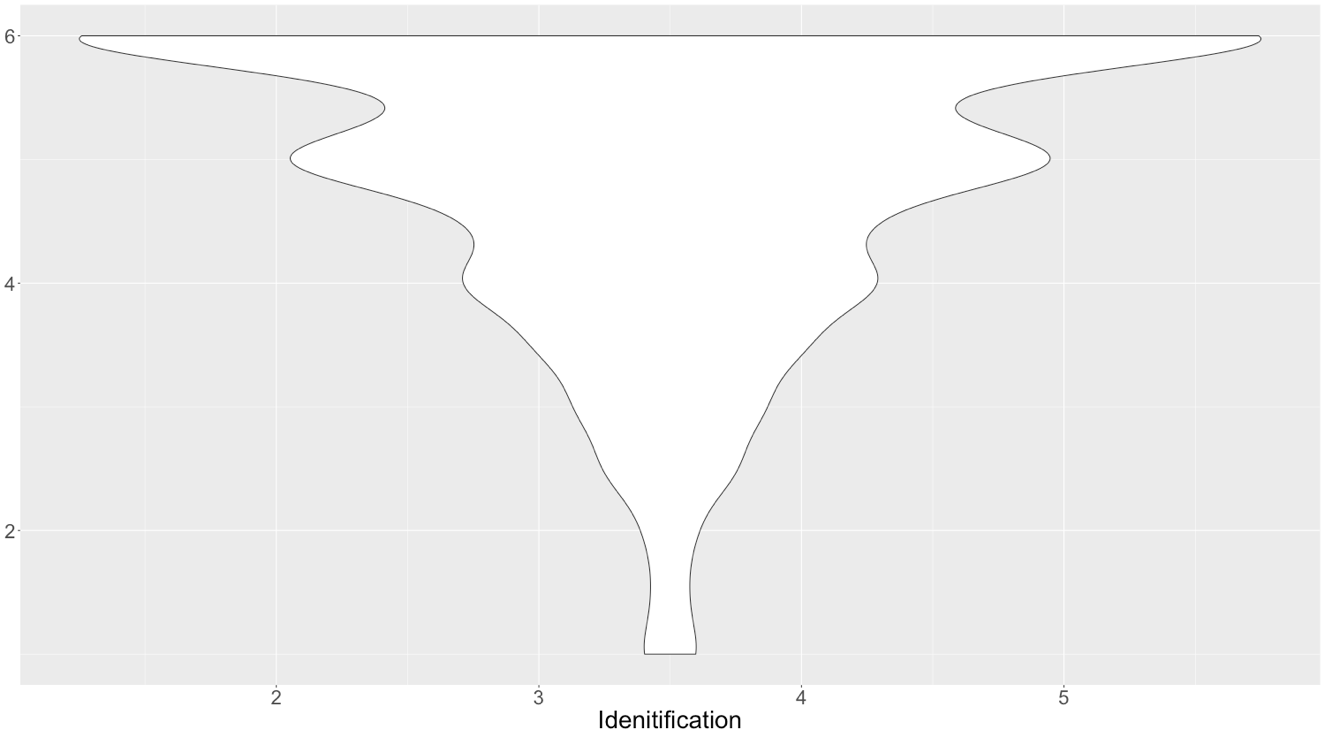


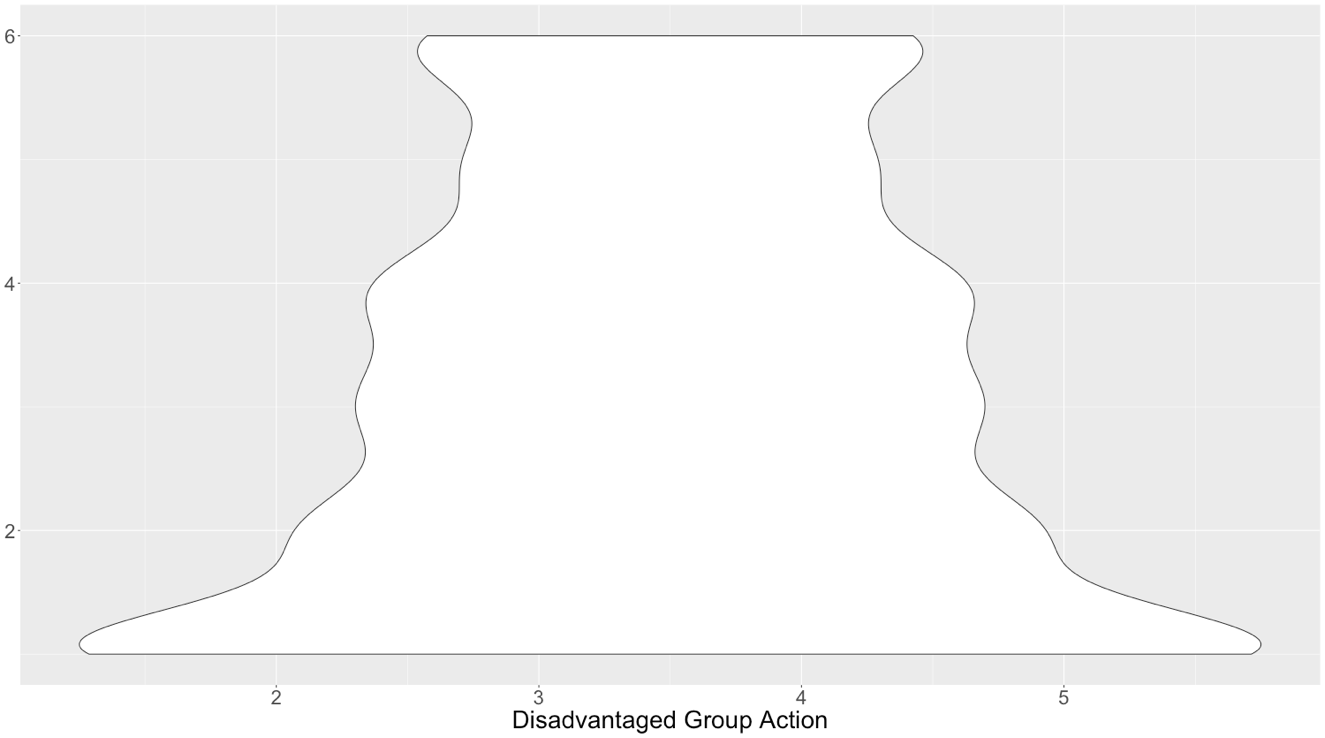


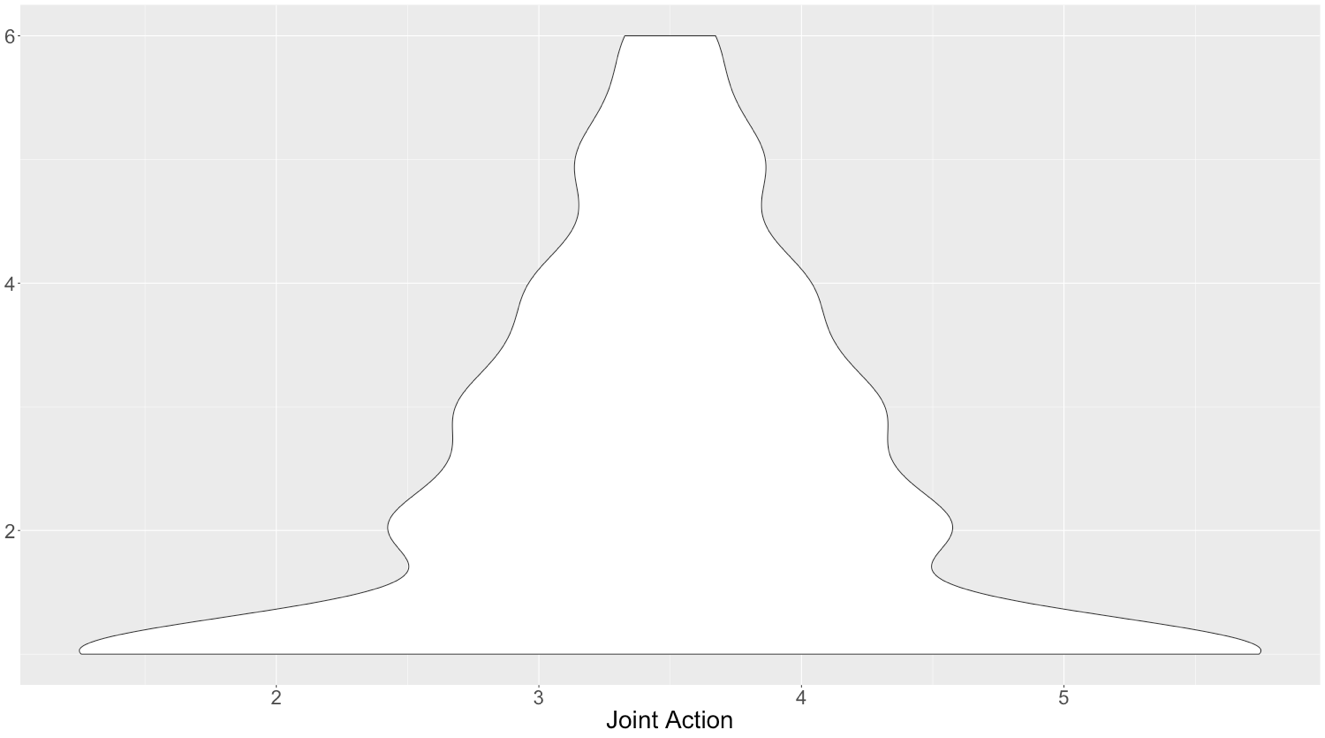


**Results when Using Activist Identification instead of Group Identification**

Table 3. Regression coefficients for the full model

|  | **Willingness to Engage in Action** | | | | |
| --- | --- | --- | --- | --- | --- |
| *Predictors* | *Estimates* | *SE* | *CI* | *Statistic* | *p* |
| Intercept | 1.09 ^***^ | 0.02 | 1.05, 1.12 | 61.00 | **<0.001** |
| Time D1 (Time 2 versus Time 1) | 0.11 ^***^ | 0.02 | 0.07, 0.15 | 4.98 | **<0.001** |
| Time D2 (Time 2 versus Time 3) | 0.01 | 0.02 | -0.03, 0.06 | 0.59 | 0.55 |
| Type of Action | 0.30 ^***^ | 0.02 | 0.25, 0.34 | 13.48 | **<0.001** |
| General Activist Identification | 0.24 ^***^ | 0.05 | 0.13, 0.34 | 4.32 | **<0.001** |
| Changes in Activist Identification | 0.12 ^***^ | 0.03 | 0.05, 0.18 | 3.55 | **<0.001** |
| Time D1 X Type of Action | -0.19 ^***^ | 0.03 | -0.25, -0.13 | -6.09 | **<0.001** |
| Time D2 X Type of Action | -0.07 ^*^ | 0.03 | -0.13, -0.01 | -2.28 | **0.02** |
| Time D1 X General Activist Identification | 0.30 ^***^ | 0.07 | 0.17, 0.44 | 4.53 | **<0.001** |
| Time D1 X General Activist Identification | 0.09 | 0.07 | -0.04, 0.22 | 1.34 | 0.18 |
| Type of Action X General Activist Identification | 0.45 ^***^ | 0.07 | 0.31, 0.58 | 6.61 | **<0.001** |
| Type of Action X Changes in Activist Identification | 0.15 ^**^ | 0.05 | 0.06, 0.24 | 3.18 | **0.001** |
| Time D1 X Type of Action X General Activist Identification | -0.51 ^***^ | 0.10 | -0.70, -0.33 | -5.37 | **<0.001** |
| Time D2 X Type of Action X General Activist Identification | -0.08 | 0.10 | -0.27, 0.11 | -0.84 | 0.40 |
| **Random Effects** | | | | | |
| σ^2^ | 0.10 | | | | |
| τ_00_ _id_ | 0.03 | | | | |
| ICC | 0.24 | | | | |
| N _id_ | 421 | | | | |
| Observations | 2502 | | | | |
| Marginal R^2^ / Conditional R^2^ | 0.252 / 0.431 | | | | |
| ** p<0.05   ** p<0.01   *** p<0.001* | | | | | |

**
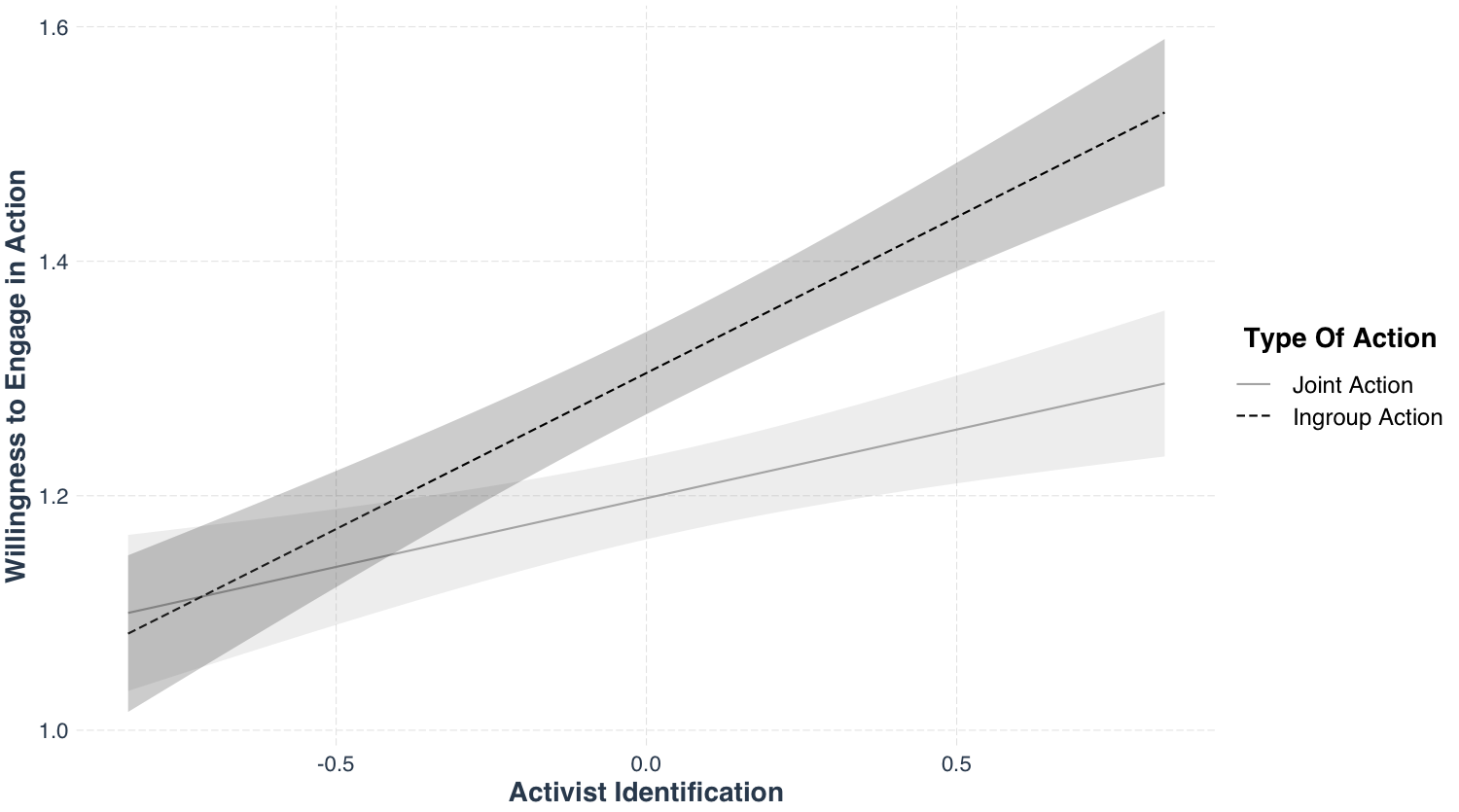
**

**Figure 7.** The interaction of change in activist identification and type of action willingness to partake in collective action across time

*Note*. Shaded areas reflect 95% confidence intervals, and all variables are log transformed.


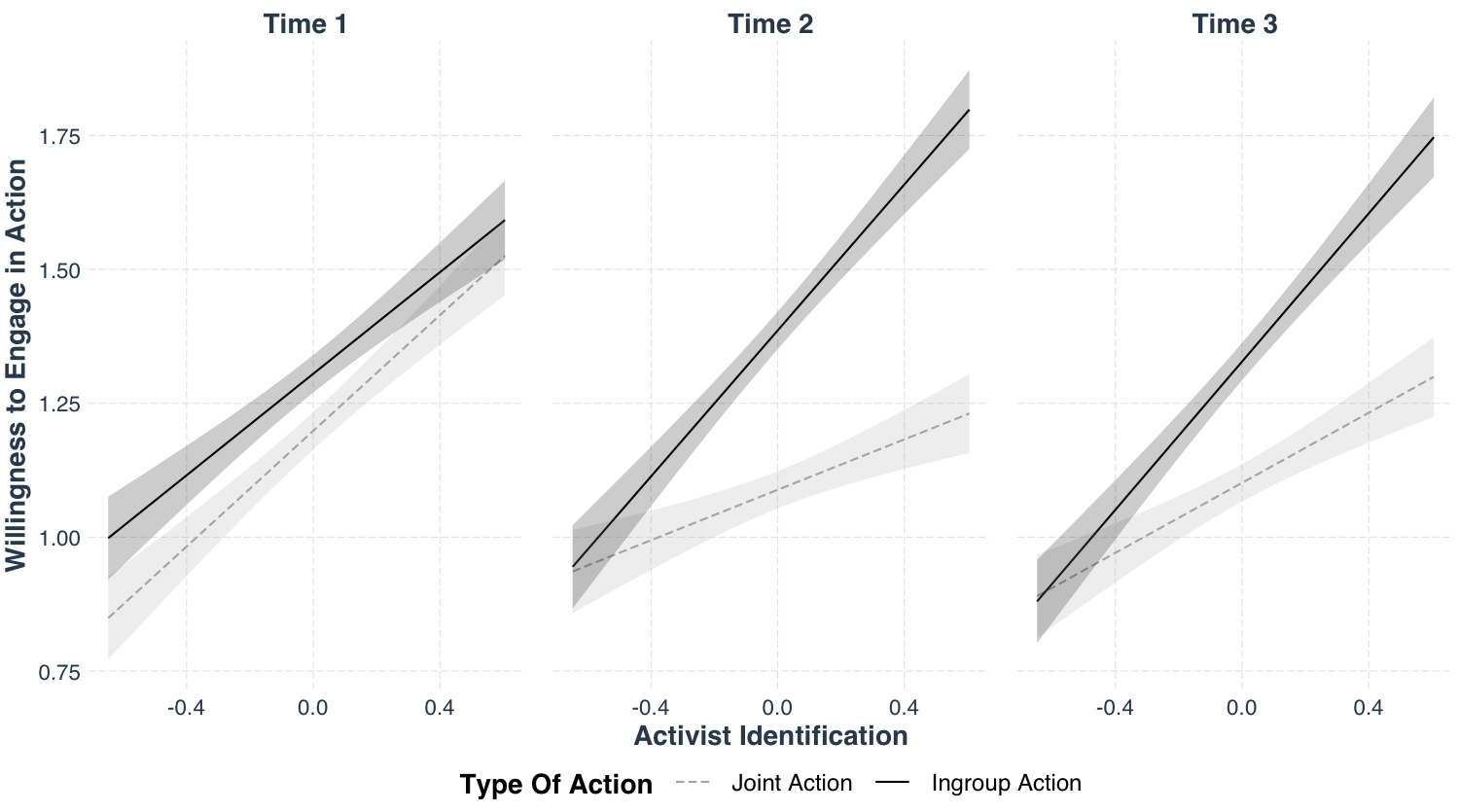


**Figure 2.** The three-way interaction of general activist identification, type of action and time on willingness to partake in collective action

*Note*. Shaded areas reflect 95% confidence intervals, and all variables are log transformed.

**Full Survey**

*Some questions were taken out from the survey after Wave 1, mostly items from multiple-item scales that displayed good reliability, and thus we could sacrifice one or two items to make room for other measures that were added in Waves 2 & 3 to allow the testing of additional research questions.*

**Perceived stability**

To what degree do you think the status of Palestinians will improve or worsen in the 5 years to come?

| (1)  Significantly worsen | (2)  Worsen | (3)  Will not change | (4)  Improve | (5)  Significantly improve |
| --- | --- | --- | --- | --- |

To what degree do you think the Israeli-Palestinian conflict will improve or worsen in the 5 years to come?

| (1)  Significantly worsen | (2)  Worsen | (3)  Will not change | (4)  Improve | (5)  Significantly improve |
| --- | --- | --- | --- | --- |

**Perceived illegitimacy**

To what extent do you agree with the following statements?

|  | (1)  I do not agree at all | (2)  I agree to almost no extent | (3)  I agree to a small extent | (4)  I agree to a moderate extent | (5)  I agree to a large extent | (6)  I agree to a very large extent |
| --- | --- | --- | --- | --- | --- | --- |
| Differences in status between Palestinians and Jewish Israelis are unfair | 1 | 2 | 3 | 4 | 5 | 6 |
| Differences in status between Palestinians and Jewish Israelis are legitimate | 1 | 2 | 3 | 4 | 5 | 6 |

**Individual mobility**

***This measure was included in Wave 1 only***

|  | (1)  Not at all | (2)  To almost no extent | (3)  To a small extent | (4)  To a moderate extent | (5)  To a large extent | (6)  To a very large extent |
| --- | --- | --- | --- | --- | --- | --- |
| To what extent do you wish to immigrate to another country | 1 | 2 | 3 | 4 | 5 | 6 |
| To what extent do you want to get an Israeli identity | 1 | 2 | 3 | 4 | 5 | 6 |
| How easy it would be for you to move somewhere else | 1 | 2 | 3 | 4 | 5 | 6 |

**Experiences of Oppression**

****This measure was added in Wave 2 and Wave 3****

Below there is a list of negative experiences. With regard to each of these experiences, please indicate to what extent you have personally suffered each of the following experiences directly or indirectly caused by the State of Israel or by any of its citizens or institutions (IDF, settlers, Israeli governmental agencies).

| (6) Always | (5) Frequently | (4) Occasionally | (3) Rarely | (2)  Ver rarely | (1)  Never |  |
| --- | --- | --- | --- | --- | --- | --- |
| 6 | 5 | 4 | 3 | 2 | 1 | House demolition or forced deportation. |
| 6 | 5 | 4 | 3 | 2 | 1 | Confiscation of land or personal property. |
| 6 | 5 | 4 | 3 | 2 | 1 | Being denied permits to visit Gaza, Jerusalem or the 1948 territories. |
| 6 | 5 | 4 | 3 | 2 | 1 | Being denied permits to visit a family member in prison. |
| 6 | 5 | 4 | 3 | 2 | 1 | Being subject to assault or threats from settlers (e.g., stabbing, shooting). |
| 6 | 5 | 4 | 3 | 2 | 1 | Being subject to mistreatment by the Israeli army at checkpoints. |
| 6 | 5 | 4 | 3 | 2 | 1 | Being subject to excessive force by the Israeli army during protests. |
| 6 | 5 | 4 | 3 | 2 | 1 | Being subject to detention, interrogation or arbitrary search by the Israeli army. |
| 6 | 5 | 4 | 3 | 2 | 1 | Having to go through checkpoints to get to school, work, etc. |
| 6 | 5 | 4 | 3 | 2 | 1 | Total shutdown of the west bank. |

***This measure was added in Wave 3***

Have you lost a close friend or family member due to the Israeli-Palestinian conflict?

1. No
2. Yes

If so, how many? _________________

**Ingroup identification**

To what extent do you agree with the following ?

|  | (1)  I do not agree at all | (2)  I agree to almost no extent | (3)  I agree to a small extent | (4)  I agree to a moderate extent | (5)  I agree to a large extent | (6)  I agree to a very large extent |
| --- | --- | --- | --- | --- | --- | --- |
| Being Palestinian is an important part of my identity. | 1 | 2 | 3 | 4 | 5 | 6 |
| I identify with other Palestinians | 1 | 2 | 3 | 4 | 5 | 6 |

**Individual emotions**

***These measures were added in Waves 2 & 3***

To what extent have you personally experiences these emotions during the past month?

|  | (1)  Not at all | (2)  To almost no extent | (3)  To a small extent | (4)  To a moderate extent | (5)  To a large extent | (6)  To a very large extent |
| --- | --- | --- | --- | --- | --- | --- |
| Humiliation | 1 | 2 | 3 | 4 | 5 | 6 |
| Anger | 1 | 2 | 3 | 4 | 5 | 6 |
| Shame | 1 | 2 | 3 | 4 | 5 | 6 |
| Pride | 1 | 2 | 3 | 4 | 5 | 6 |
| Hatred  ***Item was added in Wave 3 *** | 1 | 2 | 3 | 4 | 5 | 6 |

**Perceived capability**

****This measure was added in Wave 2 & Wave 3****

To what extent do you agree with the following?

|  | (1)  I do not agree at all | (2)  I agree to almost no extent | (3)  I agree to a small extent | (4)  I agree to a moderate extent | (5)  I agree to a large extent | (6)  I agree to a very large extent |
| --- | --- | --- | --- | --- | --- | --- |
| If I was subject to assault by the Israeli army, I think I can respond. | 1 | 2 | 3 | 4 | 5 | 6 |
| I believe there is something I can do to prevent assaults by the Israeli army. | 1 | 2 | 3 | 4 | 5 | 6 |
| I believe the Palestinian people are able to respond to assaults by the Israeli army. | 1 | 2 | 3 | 4 | 5 | 6 |
| I believe the Palestinian people are able to do something to prevent assaults by the Israeli army. | 1 | 2 | 3 | 4 | 5 | 6 |

**Identity restoration**

****This measure was added in Wave 2 & 3****

To what extent do you agree with the following?

|  | (1)  I do not agree at all | (2)  I agree to almost no extent | (3)  I agree to a small extent | (4)  I agree to a moderate extent | (5)  I agree to a large extent | (6)  I agree to a very large extent |
| --- | --- | --- | --- | --- | --- | --- |
| Resisting the Israeli occupation restores my sense of dignity. | 1 | 2 | 3 | 4 | 5 | 6 |
| Resisting the Israeli occupation gives me a sense pride about being Palestinian | 1 | 2 | 3 | 4 | 5 | 6 |
| The struggle against the Israeli occupation makes me appreciate the value of being Palestinian | 1 | 2 | 3 | 4 | 5 | 6 |

**Support of Long-term Goals and perceived progress over time**

Wave 1: To what extent do you support each of the following goals concerning the conditions of Palestinians

Wave 2: To what extent do you believe there has been progress or deterioration during the past six months in promoting the following goals concerning the conditions of Palestinians

Wave 3: To what extent do you believe there has been progress or deterioration during the past nine months in promoting the following goals concerning the conditions of Palestinians

|  | -3  Great deterioration | -2  Moderation deterioration | -1  Little deterioration | 0  No change | 1  Little progress | 2  Moderate progress | 3  Great progress |
| --- | --- | --- | --- | --- | --- | --- | --- |
| Improving the movement ability (e.g., decreasing checkpoints, issuing permits etc) | -3 | -2 | -1 | 0 | 1 | 2 | 3 |
| Issuing more working permits to Israel | -3 | -2 | -1 | 0 | 1 | 2 | 3 |
| Achieving a solution that ensures equality and democracy to everyone, both Palestinians and Jews living in historical Palestine | -3 | -2 | -1 | 0 | 1 | 2 | 3 |
| Achieving Israeli acknowledgement of and compensating Palestinians for the historical and ongoing atrocities against them | -3 | -2 | -1 | 0 | 1 | 2 | 3 |
| Fulfilling the right of return of the Palestinian refugees | -3 | -2 | -1 | 0 | 1 | 2 | 3 |
| Punishing the Jews for the historical and ongoing atrocities against Palestinians | -3 | -2 | -1 | 0 | 1 | 2 | 3 |
| Returning Jews to the countries of origin they came from | -3 | -2 | -1 | 0 | 1 | 2 | 3 |
| Establishing a Palestinian-Arab state from the river to the sea in which Jews live as second class citizens | -3 | -2 | -1 | 0 | 1 | 2 | 3 |

**Activist identity**

To what extent do you agree with the following?

|  | (1)  I do not agree at all | (2)  I agree to almost no extent | (3)  I agree to a small extent | (4)  I agree to a moderate extent | (5)  I agree to a large extent | (6)  I agree to a very large extent |
| --- | --- | --- | --- | --- | --- | --- |
| Being a political activist is an important part of my identity | 1 | 2 | 3 | 4 | 5 | 6 |
| It is important to me to view myself as a political activist. | 1 | 2 | 3 | 4 | 5 | 6 |

**Moral Obligation**

***This measure was included in Wave 1 only***

|  | (1)  Not at all | (2)  To almost no extent | (3)  To a small extent | (4)  To a certain extent | (5)  To a large extent | (6)  To a very large extent |
| --- | --- | --- | --- | --- | --- | --- |
| I feel a sense of inner obligation to actively engage in the struggle against injustice | 1 | 2 | 3 | 4 | 5 | 6 |
| I have a sense of duty toward the Palestinian cause | 1 | 2 | 3 | 4 | 5 | 6 |
| I feel a sense of responsibility toward the Palestinian society and its future | 1 | 2 | 3 | 4 | 5 | 6 |

**Empowerment**

|  | (1)  I do not agree at all | (2)  I agree to almost no extent | (3)  I agree to a small extent | (4)  I agree to a moderate extent | (5)  I agree to a large extent | (6)  I agree to a very large extent |
| --- | --- | --- | --- | --- | --- | --- |
| I am motivated to make a difference in the current situation | 1 | 2 | 3 | 4 | 5 | 6 |
| I feel that I am an active participant in, rather than an observer of, fighting against the current situation | 1 | 2 | 3 | 4 | 5 | 6 |
| I believe that doing something together is always better than doing nothing. | 1 | 2 | 3 | 4 | 5 | 6 |

**Group-based Emotions**

Wave 1: In the context of the Israeli occupation and the Palestinian-Israeli conflict, to what extent do you feel each of the following emotions:

Wave 2: In the context of the recent escalation in the West Bank and, to what extent do you feel each of the following emotions:

Wave 3: In the context of the Israeli occupation and the Palestinian-Israeli conflict, to what extent do you feel each of the following emotions:

| I feel.. | (1)  Not at all | (2)  To almost no extent | (3)  To a small extent | (4)  To a moderate extent | (5)  To a large extent | (6)  To a very large extent |
| --- | --- | --- | --- | --- | --- | --- |
| Humiliation as a result of the Israeli occupation practices | 1 | 2 | 3 | 4 | 5 | 6 |
| Hatred towards Israeli Jews | 1 | 2 | 3 | 4 | 5 | 6 |
| Anger toward the Israeli occupation | 1 | 2 | 3 | 4 | 5 | 6 |
| Contempt towards Israeli Jews | 1 | 2 | 3 | 4 | 5 | 6 |
| Anger towards the Palestinian authority | 1 | 2 | 3 | 4 | 5 | 6 |
| Despair about ending the occupation | 1 | 2 | 3 | 4 | 5 | 6 |
| Hope for ending the occupation | 1 | 2 | 3 | 4 | 5 | 6 |
| Indifference about the current situation  ***Item was included in Wave 1 only*** | 1 | 2 | 3 | 4 | 5 | 6 |
| Fear of political persecution | 1 | 2 | 3 | 4 | 5 | 6 |
| Anger towards Israeli Jews | 1 | 2 | 3 | 4 | 5 | 6 |
| Some people feel guilty about some of the violent resistance actions carried out by Palestinians. To what extent do you personally feel guilt about such actions? | 1 | 2 | 3 | 4 | 5 | 6 |
| Hope for coexistence between Palestinians and Jewish Israelis  ***Item was added in Wave 2 and Wave 3*** | 1 | 2 | 3 | 4 | 5 | 6 |
| Fear of being subject to assault by the Israeli army  ***Item was added in Wave 2 and Wave 3*** | 1 | 2 | 3 | 4 | 5 | 6 |

**Empathy**

***This measure was added in Wave 3***

Many people from different groups have suffered injury or the loss of loved ones as part of the Israeli-Palestinian conflict. To what extent do you feel empathy towards each of the following individuals who have suffered such injury or loss?

|  | (1)  I do not empathize at all | (2)  I empathize to almost no extent | (3)  I empathize to a small extent | (4)  I empathize to a moderate extent | (5)  I empathize to a large extent | (6)  I empathize to a very large extent |
| --- | --- | --- | --- | --- | --- | --- |
| Other Palestinians in the West Bank and Gaza | 1 | 2 | 3 | 4 | 5 | 6 |
| Palestinians from the 1948 territories | 1 | 2 | 3 | 4 | 5 | 6 |
| Jewish Israelis | 1 | 2 | 3 | 4 | 5 | 6 |
| Internationals visiting the country | 1 | 2 | 3 | 4 | 5 | 6 |

**Support of short-term goals**

***This measure was added in Wave 2 and Wave 3***

Wave 2: In the context of the recent escalation in the West Bank, to what extent do you support each of the following goals

Wave 3: To what extent do you support each of the following goals:

|  | (1)  I do not support at all | (2)  I support to almost no extent | (3)  I support to a small extent | (4)  I support to a moderate extent | (5)  I support to a large extent | (6)  I support to a very large extent |
| --- | --- | --- | --- | --- | --- | --- |
| Ending the tension and restoring the peace to the Palestinian streets (Wave 2).  Maintaining the peace in the Palestinian streets (Wave 3). | 1 | 2 | 3 | 4 | 5 | 6 |
| Rebel against the current situation. | 1 | 2 | 3 | 4 | 5 | 6 |
| Draining and inflicting losses on the Israeli occupation and the settlement project | 1 | 2 | 3 | 4 | 5 | 6 |
| Returning to dialogue and negotiations with Israel. | 1 | 2 | 3 | 4 | 5 | 6 |
| Taking revenge against the Israeli occupation. | 1 | 2 | 3 | 4 | 5 | 6 |
| Ending the security cooperation with Israel. | 1 | 2 | 3 | 4 | 5 | 6 |

**Power sensitivity**

To what extent do you agree with the following

|  | (1)  I do not agree at all | (2)  I agree to almost no extent | (3)  I agree to a small extent | (4)  I agree to a moderate extent | (5)  I agree to a large extent | (6)  I agree to a very large extent |
| --- | --- | --- | --- | --- | --- | --- |
| 1. It annoys me when Palestinians and Israelis are treated as if they were equally responsible for the conflict | 1 | 2 | 3 | 4 | 5 | 6 |
| 1. It annoys me that others treat the Israeli occupation as a tolerable normal situation | 1 | 2 | 3 | 4 | 5 | 6 |
| 1. It is important for me that others view the relation between Israelis and Palestinians as one between a perpetrator and a victim   ***Item was included in Wave 1 only*** | 1 | 2 | 3 | 4 | 5 | 6 |

**Moral convictions**

|  | (1)  I do not agree at all | (2)  I agree to almost no extent | (3)  I agree to a small extent | (4)  I agree to a moderate extent | (5)  I agree to a large extent | (6)  I agree to a very large extent |
| --- | --- | --- | --- | --- | --- | --- |
| 1. My opinion about the Israeli-Palestinian conflict reflects an important part of who I am | 1 | 2 | 3 | 4 | 5 | 6 |
| 1. My opinion about the Israeli-Palestinian conflict is an important part of my moral norms and values | 1 | 2 | 3 | 4 | 5 | 6 |

**Perceived identity of subgroup**

It can be said that Arabs with Israeli citizenship are related to both to Israelis and Palestinians. To what extent do you agree with that.

To what extent are they Israelis and to what extent are they Palestinians (1= not at all, 100 = to a very large extent).

- Israelis (1-100)
- Palestinians (1-100)

**Perceived network emotions**

Wave 1: Try to think about the 10 closest people to you. It can be family or friends.

To what extent do you think these close people feel each of the following emotions

Wave 2: Try to think about the 10 closest people to you. It can be family or friends.

To what extent do you think these close people feel each of the following emotions in the context of the recent events in the West Bank

Wave 3: Try to think about the 10 closest people to you. It can be family or friends.

To what extend do you think these close people feel each of the following emotions

| They feel… | (1)  Not at all | (2)  To almost no extent | (3)  To a small extent | (4)  To a moderate extent | (5)  To a large extent | (6)  To a very large extent |
| --- | --- | --- | --- | --- | --- | --- |
| Anger towards Israeli Jews | 1 | 2 | 3 | 4 | 5 | 6 |
| Hatred towards Israeli Jews | 1 | 2 | 3 | 4 | 5 | 6 |
| Anger toward the Israeli occupation. | 1 | 2 | 3 | 4 | 5 | 6 |
| Contempt towards Israeli Jews | 1 | 2 | 3 | 4 | 5 | 6 |
| Anger towards the Palestinian authority | 1 | 2 | 3 | 4 | 5 | 6 |
| Despair about ending the occupation | 1 | 2 | 3 | 4 | 5 | 6 |
| Hope for ending the occupation | 1 | 2 | 3 | 4 | 5 | 6 |
| Fear of political persecution. | 1 | 2 | 3 | 4 | 5 | 6 |
| Humiliation as a result of the Israeli occupation practices | 1 | 2 | 3 | 4 | 5 | 6 |
| Some people feel guilty about some of the violent resistance actions carried out by Palestinians. To what extend do you think they feel guilt about such actions? | 1 | 2 | 3 | 4 | 5 | 6 |
| Hope for coexistence between Palestinians and Jewish Israelis  ***Item was added in Wave 2 & 3*** | 1 | 2 | 3 | 4 | 5 | 6 |
| Fear of being subject to assault by the Israeli army  ***Item was added in Wave 2 & 3*** | 1 | 2 | 3 | 4 | 5 | 6 |

**Perceived attitudes similarity with network**

To what extent do you share the same political approaches with these people

| (1)  Not at all | (2)  To almost no extent | (3)  To a small extent | (4)  To a moderate extent | (5)  To a large extent | (6)  To a very large extent |
| --- | --- | --- | --- | --- | --- |

To what extent do you share you opinion regarding major Palestinian issues with these people (Refugees, East Jerusalem).

| (1)  Not at all | (2)  To almost no extent | (3)  To a small extent | (4)  To a moderate extent | (5)  To a large extent | (6)  To a very large extent |
| --- | --- | --- | --- | --- | --- |

**Embeddedness in activist network**

How many of these close people are activists that engage in demonstrations and political activities?

(0) None (1) One (2) Two (3) Three (4) Four (5) Five or more

If there is 1 activist or more:

- 1. How much time do you spend with these activists in your network?

| (1)  Never | (2)  Rarely | (3)  Sometimes | (4)  Often | (5)  All the time |
| --- | --- | --- | --- | --- |

- 1. How often do these activists in your network ask you to join protests or other political activities?

| (1)  Never | (2)  Rarely | (3)  Sometimes | (4)  Often | (5)  All the time |
| --- | --- | --- | --- | --- |

- 1. How important is it for you to be in contact with these activists friends?

| (1)  Not Important At All | (2)  Slightly Important | (3)  Moderately Important | (4)  Important | (5)  Very important |
| --- | --- | --- | --- | --- |

**Distinctiveness threat**

To what extent do you agree with the following sentences regarding 1948 Arabs (1=not at all, 6=to a large extent).

- It annoys me when others don’t see the difference between 48 Arabs and west bank Arabs.
- It’s not right when 48 Arabs and west bank Arabs are treated the same way.

**Expectations from the ingroup**

What do you think will happen if the occupation ends one day?

|  | (1)  I do not agree at all | (2)  I agree to almost no extent | (3)  I agree to a small extent | (4)  I agree to a moderate extent | (5)  I agree to a large extent | (6)  I agree to a very large extent |
| --- | --- | --- | --- | --- | --- | --- |
| I expect that Palestinians will remain internally divided | 1 | 2 | 3 | 4 | 5 | 6 |
| I expect that Palestinians will be capable of establishing a democratic society | 1 | 2 | 3 | 4 | 5 | 6 |
| I expect that Palestinians will progress as a society in education, science and economics | 1 | 2 | 3 | 4 | 5 | 6 |

**Expectations from the outgroup**

|  | (1)  I do not agree at all | (2)  I agree to almost no extent | (3)  I agree to a small extent | (4)  I agree to a moderate extent | (5)  I agree to a large extent | (6)  I agree to a very large extent |
| --- | --- | --- | --- | --- | --- | --- |
| I expect that Israeli Jews’ support for the occupation will decrease with time (Wave 1)  During the past *six* month that Israeli Jews’ support for the occupation has decreased (Wave 2)  During the past *nine* month that Israeli Jews’ support for the occupation has decreased (Wave 3) | 1 | 2 | 3 | 4 | 5 | 6 |
| I expect that with time Israeli Jews will become aware of the injustice they committed against Palestinians (Wave 1)  During the past *six* months Israeli Jews have become aware of the injustice they committed against Palestinians (Wave 2)  During the past *nine* months Israeli Jews have become aware of the injustice they committed against Palestinians (Wave 3) | 1 | 2 | 3 | 4 | 5 | 6 |
| I think that sooner or later Israeli Jews will start to change (positively) their attitudes and stances toward Palestinians (Wave 1)  During the past *six* month Israeli Jews have started to change (positively) their attitudes and stances toward Palestinians (Wave 2)  During the past *nine* month Israeli Jews have started to change (positively) their attitudes and stances toward Palestinians (Wave 3) | 1 | 2 | 3 | 4 | 5 | 6 |

**Expectations from ingroup leadership**

***Measure was included in Wave 1 only***

|  | (1)  Not at all | (2)  To almost no extent | (3)  To a small extent | (4)  To a moderate extent | (5)  To a large extent | (6)  To a very large extent |
| --- | --- | --- | --- | --- | --- | --- |
| I expect that sooner or later, the Palestinian authority will end its cooperation with Israel | 1 | 2 | 3 | 4 | 5 | 6 |
| I expect that the Palestinian authority will achieve an independent Palestinian state | 1 | 2 | 3 | 4 | 5 | 6 |

**Perceived ingroup emotions**

Wave 1: In the context of the Palestinian-Israeli conflict and the Israeli occupation, to what extent do you think Palestinians in general experience each of the following emotions

Wave 2: In the context of the recent escalation in the West Bank, to what extent do you think Palestinians in general experience each of the following emotions

Wave 3: In the context of the Palestinian-Israeli conflict and the Israeli occupation, to what extent do you think Palestinians in general experience each of the following emotions

| Palestinians feel… | (1)  Not at all | (2)  To almost no extent | (3)  To a small extent | (4)  To a moderate extent | (5)  To a large extent | (6)  To a very large extent |
| --- | --- | --- | --- | --- | --- | --- |
| Anger towards Israeli Jews | 1 | 2 | 3 | 4 | 5 | 6 |
| Hatred towards Israeli Jews | 1 | 2 | 3 | 4 | 5 | 6 |
| Anger toward the Israeli occupation. | 1 | 2 | 3 | 4 | 5 | 6 |
| Contempt towards Israeli Jews | 1 | 2 | 3 | 4 | 5 | 6 |
| Anger towards the Palestinian authority | 1 | 2 | 3 | 4 | 5 | 6 |
| Despair about ending the occupation | 1 | 2 | 3 | 4 | 5 | 6 |
| Hope for ending the occupation | 1 | 2 | 3 | 4 | 5 | 6 |
| Fear of political persecution. | 1 | 2 | 3 | 4 | 5 | 6 |
| Humiliation as a result of the Israeli occupation practices | 1 | 2 | 3 | 4 | 5 | 6 |
| Some people feel guilty about some of the violent resistance actions carried out by Palestinians. To what extend do you think they feel guilt about such actions? | 1 | 2 | 3 | 4 | 5 | 6 |
| Hope for coexistence between Palestinians and Jewish Israelis  ***Item was added in Wave 2 & 3*** | 1 | 2 | 3 | 4 | 5 | 6 |
| Fear of being subject to assault by the Israeli army  ***Item was added in Wave 2 & 3*** | 1 | 2 | 3 | 4 | 5 | 6 |

**Realistic and symbolic threat**

To what extent do you agree with the following sentences regarding 48 Arabs (1=not at all, 6=to a very large extent).

- In general there is resemblances in traditions between 48 Arabs and Jews in Israel.
- 48 Arabs are responsible for cultural regressions’(Arab or Palestinian culture).
- 48 Arabs use up all the local resources on the expense of Jews
- 48 Arabs pose a threat to the safety of Palestinians in the west bank

**Essentialism**

- To what extent do you agree with the following sentences (1=not at all, 6=to a very large extent).
- If you identify with a certain social group, you cannot identify with another.
- There are different types of social groups and you can categorize them simply and clearly.

**Basic needs**

How satisfied are you with the following things?

|  | (1)  Not at all | (2)  To almost no extent | (3)  To a small extent | (4)  To a moderate extent | (5)  To a large extent | (6)  To a very large extent |
| --- | --- | --- | --- | --- | --- | --- |
| The amount of sleep you get to feel refreshed | 1 | 2 | 3 | 4 | 5 | 6 |
| Overall physical strength | 1 | 2 | 3 | 4 | 5 | 6 |
| Access to health services | 1 | 2 | 3 | 4 | 5 | 6 |
| Socio-economic condition | 1 | 2 | 3 | 4 | 5 | 6 |
| Physical safety (from occupation) | 1 | 2 | 3 | 4 | 5 | 6 |

**Motivation for emotions**

Imagine you could have perfect control over your emotions. To what extent would you want to feel the following emotions?

| I want to feel.. | (1)  Not at all | (2)  To almost no extent | (3)  To a small extent | (4)  To a moderate extent | (5)  To a large extent | (6)  To a very large extent |
| --- | --- | --- | --- | --- | --- | --- |
| Hope for ending the occupation | 1 | 2 | 3 | 4 | 5 | 6 |
| Hope for coexistence between Palestinians and Jewish Israelis | 1 | 2 | 3 | 4 | 5 | 6 |
| Anger or more anger about the occupation | 1 | 2 | 3 | 4 | 5 | 6 |
| Anger or more anger towards Jewish Israelis | 1 | 2 | 3 | 4 | 5 | 6 |
| Pride of the Palestinian people | 1 | 2 | 3 | 4 | 5 | 6 |

**Short-term hope and hopelessness**

To what extent do you agree with the following

|  | (1)  I do not agree at all | (2)  I agree to almost no extent | (3)  I agree to a small extent | (4)  I agree to a moderate extent | (5)  I agree to a large extent | (6)  I agree to a very large extent |
| --- | --- | --- | --- | --- | --- | --- |
| I do not think there is a chance that the occupation will end in the near future | 1 | 2 | 3 | 4 | 5 | 6 |
| I imagine that the coming few years will bring more tragedies on the Palestinian people | 1 | 2 | 3 | 4 | 5 | 6 |
| I foresee signs of improvement in the Palestinian situation in the near future. | 1 | 2 | 3 | 4 | 5 | 6 |
| I think that the onset of the occupation dismantlement is possible in the near future | 1 | 2 | 3 | 4 | 5 | 6 |

**Long-term hope and hopelessness**

|  | (1)  I do not agree at all | (2)  I agree to almost no extent | (3)  I agree to a small extent | (4)  I agree to a moderate extent | (5)  I agree to a large extent | (6)  I agree to a very large extent |
| --- | --- | --- | --- | --- | --- | --- |
| Even if the Palestinian condition won’t improve in the near future, I believe that my grandchildren and great-grandchildren will live the liberation from the occupation | 1 | 2 | 3 | 4 | 5 | 6 |
| I think the Israeli occupation will end in the near or far future | 1 | 2 | 3 | 4 | 5 | 6 |
| I think it’s impossible to achieve peace with Jewish Israelis some day | 1 | 2 | 3 | 4 | 5 | 6 |
| Its hard to imagine that Palestinians will ever have freedom | 1 | 2 | 3 | 4 | 5 | 6 |
| I think it is impossible that injustice will come to an end someday  ***Items added in Waves 2 & 3*** | 1 | 2 | 3 | 4 | 5 | 6 |

**Wishes and Expectations**

***These measures were included in Wave 3 only***

In the next questions, we are interested to learn only about your **wishes for the future.**

Please indicate how much do you **wish** the following propositions to materialize?

(1= have no specific wish, 6= wish very much).

|  | **6**  **I Wish very much** | **5** | **4** | **3** | **2** | **1**  **I have no such wish** |
| --- | --- | --- | --- | --- | --- | --- |
| Ending the Palestinian-Israeli conflict | 6 | 5 | 4 | 3 | 2 | 1 |
| Ending the conflict in a way that addresses the core needs of both peoples. | 6 | 5 | 4 | 3 | 2 | 1 |
| Ending the conflict in a way that assures independence and freedom for Palestinians and security and safety for Israelis. | 6 | 5 | 4 | 3 | 2 | 1 |

Now, we are interested to learn only about your **expectations** pertaining to the future of the conflict.

Please indicate how much you **expect** the following proposition to **actually materialize**?

(1 = no likelihood whatsoever to 6 = very high likelihood)

|  | **6**  **Very Likely** | **5** | **4** | **3** | **2** | **1**  **Very**  **Unlikely** |
| --- | --- | --- | --- | --- | --- | --- |
| Ending the Palestinian-Israeli conflict | 6 | 5 | 4 | 3 | 2 | 1 |
| Ending the conflict in a way that addresses the core needs of both peoples. | 6 | 5 | 4 | 3 | 2 | 1 |
| Ending the conflict in a way that assures independence and freedom for Palestinians and security and safety for Israelis. | 6 | 5 | 4 | 3 | 2 | 1 |

**Group Efficacy Beliefs**

|  | (1)  I do not agree at all | (2)  I agree to almost no extent | (3)  I agree to a small extent | (4)  I agree to a moderate extent | (5)  I agree to a large extent | (6)  I agree to a very large extent |
| --- | --- | --- | --- | --- | --- | --- |
| I believe that we Palestinians, as a group, can achieve our goals | 1 | 2 | 3 | 4 | 5 | 6 |
| I believe that we Palestinians, together, can end occupation | 1 | 2 | 3 | 4 | 5 | 6 |
| I believe that we Palestinians, together, can win the struggle against occupation  ***Item included in Wave 1 only*** | 1 | 2 | 3 | 4 | 5 | 6 |

**Perceptions of subgroup as a gateway community**

To what extent do you agree with the following, regarding 48 Arabs (1=not at all, 6=to a very large extent).

48 Arabs are:

- Middle ground between Israelis and Palestinians.
- A linking part between the Arabic world and Israel.
- They aren’t a loyal group and can betray Palestinians in case of a war with Israel.
- A group with special qualities than can lead a political change.
- A weak group that cannot benefit itself nor others.

**Perceived appropriateness of emotions**

***This measure was added in Waves 2 and 3***

In the context of the Israeli occupation and its practices in the West Bank…

|  | (1)  Not at all | (2)  To almost no extent | (3)  To a small extent | (4)  To a moderate extent | (5)  To a large extent | (6)  To a very large extent |
| --- | --- | --- | --- | --- | --- | --- |
| I think that Palestinians should feel despair about the occupation. | 1 | 2 | 3 | 4 | 5 | 6 |
| I think that Palestinians should feel humiliation as a result of the occupation. | 1 | 2 | 3 | 4 | 5 | 6 |
| I think that Palestinians should feel anger toward the Israeli occupation | 1 | 2 | 3 | 4 | 5 | 6 |
| I think that Palestinians should feel hatred towards the Israeli occupation | 1 | 2 | 3 | 4 | 5 | 6 |
| I think that Palestinians should feel hope for ending the occupation | 1 | 2 | 3 | 4 | 5 | 6 |

**Perceived outgroup homogeneity**

To what extent do you agree with the following:

| Jewish Israelis are similar to each other in their attitudes toward Palestinians | 1 | 2 | 3 | 4 | 5 | 6 |
| --- | --- | --- | --- | --- | --- | --- |
| Jewish Israelis are similar to each other in their morals | 1 | 2 | 3 | 4 | 5 | 6 |
| Jewish Israelis are similar to each other in their personal characteristics | 1 | 2 | 3 | 4 | 5 | 6 |

**Motivation for outgroup emotions**

Wave 1: Imagine you have complete control over how Jewish Israelis feel, to what extent do you want them to feel the following emotions in the context of the Israeli occupation:

Wave 2: Imagine you have complete control over how Jewish Israelis feel, to what extent do you want them to feel the following emotions in the context of the recent escalation in the West Bank

Wave 3: Imagine you have complete control over how Jewish Israelis feel, to what extent do you want them to feel the following emotions in the context of the Israeli occupation:

| *I want Israeli Jews to feel..* | (1)  Not at all | (2)  To almost no extent | (3)  To a small extent | (4)  To a moderate extent | (5)  To a large extent | (6)  To a very large extent |
| --- | --- | --- | --- | --- | --- | --- |
| Guilt over the occupation | 1 | 2 | 3 | 4 | 5 | 6 |
| Regret over the occupation | 1 | 2 | 3 | 4 | 5 | 6 |
| Shame about the occupation | 1 | 2 | 3 | 4 | 5 | 6 |
| Anger about the occupation | 1 | 2 | 3 | 4 | 5 | 6 |
| Fear of Palestinians | 1 | 2 | 3 | 4 | 5 | 6 |
| Hope for peace | 1 | 2 | 3 | 4 | 5 | 6 |
| I do not care what Israeli Jews feel  ***Item included in Wave 1 only*** | 1 | 2 | 3 | 4 | 5 | 6 |

**Collective Action**

The following questions address you opinion regarding peaceful and non-peaceful and resistance.

Peaceful resistance, by its definition, means resisting without the usage of violent methods.

Non-peaceful resistance is based on using force and violent methods such as armed resistance, throwing rocks and more.

**Perceived effectiveness of non-violent action**

To what extent do you agree with the following:

|  | (1)  I do not agree at all | (2)  I agree to almost no extent | (3)  I agree to a small extent | (4)  I agree to a moderate extent | (5)  I agree to a large extent | (6)  I agree to a very large extent |
| --- | --- | --- | --- | --- | --- | --- |
| Mass non-violent action puts pressure on Israel while also undermining its excuse that peace cannot be achieved as long as there is violence against Israelis | 1 | 2 | 3 | 4 | 5 | 6 |
| Nonviolent action can be effective at achieving the goals of Palestinians | 1 | 2 | 3 | 4 | 5 | 6 |

**Perceived effectiveness of violent action**

|  | (1)  I do not agree at all | (2)  I agree to almost no extent | (3)  I agree to a small extent | (4)  I agree to a moderate extent | (5)  I agree to a large extent | (6)  I agree to a very large extent |
| --- | --- | --- | --- | --- | --- | --- |
| Using non-peaceful resistance increases the likelihood that Israel will comply with the Palestinian demands. | 1 | 2 | 3 | 4 | 5 | 6 |
| Non-peaceful methods are effective in attracting extensive media attention to the Palestinian cause | 1 | 2 | 3 | 4 | 5 | 6 |

**Tolerance of non-violent and joint collective action**

Wave 1: In the context of the ongoing struggle against the Israeli occupation, to what extent do you understand people who take part in these actions

Please note that the questions do not address your own actions**.**

Wave 2: In the context of the recent protests against the Israeli occupation, to what extent do you understand people who take part in these actions

Wave 3: In the context of the ongoing struggle against the Israeli occupation, to what extent do you understand people who take part in these actions.

|  | (1)  Not at all | (2)  To almost no extent | (3)  To a small extent | (4)  To a moderate extent | (5)  To a large extent | (6)  To a very large extent |
| --- | --- | --- | --- | --- | --- | --- |
| Participating in peaceful demonstrations against the occupation | 1 | 2 | 3 | 4 | 5 | 6 |
| Participating in sit-ins against occupation  ***item Included in Wave 1 only*** | 1 | 2 | 3 | 4 | 5 | 6 |
| Acting within peaceful social political movements against the occupation | 1 | 2 | 3 | 4 | 5 | 6 |
| Participating in joint Palestinian-Israeli peace initiatives | 1 | 2 | 3 | 4 | 5 | 6 |
| Participating in joint Palestinian-Israeli demonstrations against the occupation | 1 | 2 | 3 | 4 | 5 | 6 |

**Tolerance of violent collective action**

| To what extent do you think it is understandable that people resort to non-peaceful | (1)  Not justified at all | (2)  Justified to almost no extent | (3)  Justified to a small extent | (4)  Justified to a moderate extent | (5)  Justified to a large extent | (6)  Justified to a very large extent |
| --- | --- | --- | --- | --- | --- | --- |
| To what extent do you think it is legitimate that people resort to non-peaceful | (1)  Not legitimate at all | (2)  Legitimate to almost no extent | (3)  Legitimate to a small extent | (4)  Legitimate to a moderate extent | (5)  Legitimate to a large extent | (6)  Legitimate to a very large extent |
| To what extent do you understand the reasons that lead people to engage in non-peaceful resistance  ***Item included in Wave 1 only*** | (1)  I do not understand at all | (2)  I understand to almost no extent | (3)  I understand to a small extent | (4)  I understand to a moderate extent | (5)  I understand to a large extent | (6)  I understand to a very large extent |
| To what extent do you understand people wo engage in confrontations with the Israeli army  ***Item included in Wave 1 only*** | (1)  I do not understand at all | (2)  I understand to almost no extent | (3)  I understand to a small extent | (4)  I understand to a moderate extent | (5)  I understand to a large extent | (6)  I understand to a very large extent |
| To what extent do you understand people who engage in armed resistance. | (1)  I do not understand at all | (2)  I understand to almost no extent | (3)  I understand to a small extent | (4)  I understand to a moderate extent | (5)  I understand to a large extent | (6)  I understand to a very large extent |

**Perceived instrumentality of action**

***Measure was included in Wave 2 only***

To what degree do you believe each of the following actions can achieve positive results concerning the Palestinian struggle for liberation

|  | (1)  Does not achieve at all | (2)  Achieves to almost no extent | (3)  Achieves to a small extent | (4)  Achieves to a moderate extent | (5)  Achieves to a large extent | (6)  Achieves to a very large extent |
| --- | --- | --- | --- | --- | --- | --- |
| Armed resistance | 1 | 2 | 3 | 4 | 5 | 6 |
| Popular peaceful struggle | 1 | 2 | 3 | 4 | 5 | 6 |
| The collective action for boycott, divestment, sanctions on Israel | 1 | 2 | 3 | 4 | 5 | 6 |

**Collective action tendencies**

Wave 1: In the context of the ongoing struggle against the Israeli occupation, to what degree are YOU personally willing to engage in the following actions:

Wave 2: In the context of the recent protests against the Israeli occupation, to what degree are YOU personally willing to engage in the following actions:

Wave 3: In the context of the ongoing struggle against the Israeli occupation, to what degree are YOU personally willing to engage in the following actions:

|  | (1)  Not at all | (2)  To almost no extent | (3)  To a small extent | (4)  To a moderate extent | (5)  To a large extent | (6)  To a very large extent |
| --- | --- | --- | --- | --- | --- | --- |
| Participating in peaceful demonstrations against the occupation | 1 | 2 | 3 | 4 | 5 | 6 |
| Participating in peaceful sit-ins against the occupation | 1 | 2 | 3 | 4 | 5 | 6 |
| Acting within peaceful social political movements against occupation | 1 | 2 | 3 | 4 | 5 | 6 |
| Supporting the political and economic boycott of Israel | 1 | 2 | 3 | 4 | 5 | 6 |
| Organizing campaigns targeting the public and merchants to boycott Israeli products  **Item was added to Wave 2 & 3*** | 1 | 2 | 3 | 4 | 5 | 6 |
| Participating in demonstrations that could involve confrontations with the Israeli army | 1 | 2 | 3 | 4 | 5 | 6 |
| Supporting non-peaceful resistance. | 1 | 2 | 3 | 4 | 5 | 6 |

**Willingness to compromise**

|  | (1)  I do not support at all | (2)  I support to almost no extent | (3)  I support to a small extent | (4)  I support to a moderate extent | (5)  I support to a large extent | (6)  I support to a very large extent |
| --- | --- | --- | --- | --- | --- | --- |
| Supposedly it was feasible, to what extent would you support a peace agreement for two states based on the 1967 borders, with (only) *east* Jerusalem as the capital of Palestine? | 1 | 2 | 3 | 4 | 5 | 6 |
| Supposedly it was feasible, to what extent would you support a resolution of two states based on the 1967 borders that includes land exchange instead of dismantlement of the settlements? | 1 | 2 | 3 | 4 | 5 | 6 |
| Supposedly it was feasible, to what extent would you support a resolution of two states based on the 1967 borders, without the return of the refugees? | 1 | 2 | 3 | 4 | 5 | 6 |
| Supposedly it was feasible, to what extent would you support a one democratic state solution for both Palestinians and Jews? | 1 | 2 | 3 | 4 | 5 | 6 |

|  | (1)  Not at all | (2)  To almost no extent | (3)  To a small extent | (4)  To a moderate extent | (5)  To a large extent | (6)  To a very large extent |
| --- | --- | --- | --- | --- | --- | --- |
| Participating in joint Palestinian-Israeli peace initiatives | 1 | 2 | 3 | 4 | 5 | 6 |
| Participating in joint Palestinian-Israeli demonstrations against the occupation | 1 | 2 | 3 | 4 | 5 | 6 |

**Beliefs about sacrifice**

***This measure was added to Wave 3***

To what extent do you agree with the following?

|  | (1)  I do not agree at all | (2)  I agree to almost no extent | (3)  I agree to a small extent | (4)  I agree to a moderate extent | (5)  I agree to a large extent | (6)  I agree to a very large extent |
| --- | --- | --- | --- | --- | --- | --- |
| The struggle for freedom and justice demands sacrifice from every Palestinian | 1 | 2 | 3 | 4 | 5 | 6 |
| Any Palestinian harmed by Jewish Israelis, including one who was not involved in fighting, is a freedom fighter whose suffering serves the Palestinian cause | 1 | 2 | 3 | 4 | 5 | 6 |
| All resistance to the Israeli occupation is legitimate, even if it Jewish Israelis who are not directly involved in fighting are harmed | 1 | 2 | 3 | 4 | 5 | 6 |

**Past activism**

|  | (1)  I never participated | (2)  I rarely participated | (3)  I participated occasionally | (4)  I moderately participated | (5)  I often participated | (6)  I participated all the time |
| --- | --- | --- | --- | --- | --- | --- |
| To what degree were you involved in political activism during the past 5 years | 1 | 2 | 3 | 4 | 5 | 6 |

**Burnout**

*If the answer to the previous question was 1, please skip this part.*

|  | (1)  I do not agree at all | (2)  I agree to almost no extent | (3)  I agree to a small extent | (4)  I agree to a moderate extent | (5)  I agree to a large extent | (6)  I agree to a very large extent |
| --- | --- | --- | --- | --- | --- | --- |
| I feel drained as part of being politically active. | 1 | 2 | 3 | 4 | 5 | 6 |
| I feel mentally stressed as part of being politically active. | 1 | 2 | 3 | 4 | 5 | 6 |

**Past Activism**

***This measure was added to Wave 3***

To what degree have you been involved in these actions in the past **9 months**

|  | (1)  Not at all | (2)  To almost no extent | (3)  To a small extent | (4)  To a certain extent | (5)  To a large extent | (6)  To a very large extent |
| --- | --- | --- | --- | --- | --- | --- |
| Participating in peaceful demonstrations against occupation | 1 | 2 | 3 | 4 | 5 | 6 |
| Participating in peaceful sit-ins against occupation | 1 | 2 | 3 | 4 | 5 | 6 |
| Acting within peaceful social political movements against occupation | 1 | 2 | 3 | 4 | 5 | 6 |
| Supporting the political and economic boycott of Israel | 1 | 2 | 3 | 4 | 5 | 6 |
| Organizing campaigns targeting the public and merchants to boycott Israeli products | 1 | 2 | 3 | 4 | 5 | 6 |
| Participating in demonstrations that could involve confrontations with the Israeli army | 1 | 2 | 3 | 4 | 5 | 6 |
| Supporting non-peaceful resistance. | 1 | 2 | 3 | 4 | 5 | 6 |
| Participating in joint Palestinian-Israeli peace initiatives | 1 | 2 | 3 | 4 | 5 | 6 |
| Participating in joint Palestinian-Israeli demonstrations against the occupation | 1 | 2 | 3 | 4 | 5 | 6 |

**Barriers to collective action**

To what extent does each of the following things hinder your ability to take part in political activism? Order them from the most influential to the least (from 1-Most influential to 5-Least influential).

- - Family commitments
  - Work commitments
  - Academic commitments
  - Financial sacrifices
  - Geographic distance

**Perceived risks of collective action**

Which of the following threats do take in considerations in the decision to engage in collective action?

|  | (1)  Not at all | (2)  To almost no extent | (3)  To a small extent | (4)  To a moderate extent | (5)  To a large extent | (6)  To a very large extent |
| --- | --- | --- | --- | --- | --- | --- |
| Political prosecutions | 1 | 2 | 3 | 4 | 5 | 6 |
| Physical trauma | 1 | 2 | 3 | 4 | 5 | 6 |
| Losing job | 1 | 2 | 3 | 4 | 5 | 6 |
| Potential hurt to family | 1 | 2 | 3 | 4 | 5 | 6 |
| Jail | 1 | 2 | 3 | 4 | 5 | 6 |

**Demographics (Wave 1)**

- **Age_t1**
- **Gender_t1**

1. Male
2. Female

- **Education_t1**

1. School diploma or lower
2. Certificate
3. Firs degree
4. Second degree or higher

- **Residence_t1**

1. Village
2. City
3. Refugee camp

- **Religion_t1**

1. Muslim
2. Christian
3. Other

- **Religious_t1**

1. Secular
2. Secular with conservative tendencies
3. Conservative
4. Religious
5. Extremely Religious

- **Profession_t1**

1. Governmental employee
2. Academic
3. Journalism/media
4. Private sector
5. Civil organization
6. Political leadership
7. Unemployed
8. Driver
9. Other

- **Family_t1**

Number of family members

- **Income_t1**

1. 5000 NIS or higher

2. 4500 - 4999

3. 4000 -4499

4. 3500 - 3999

5. 3000 - 3499

6. 2500 - 2999

7. 2000 - 2499

8. 1500 - 1999

9. 1000 - 1499

10. 500 - 999

11. less than 500

99. I don’t know/ no response

- **PrisonWent_t1**

1. Yes
2. No

- **Prison_2_t1**

Prison duration
